# The Epidemiology of Alcohol Use and Alcohol Use Disorders among Young People in Northern Tanzania

**DOI:** 10.1371/journal.pone.0140041

**Published:** 2015-10-07

**Authors:** Joel M. Francis, Helen A. Weiss, Gerry Mshana, Kathy Baisley, Heiner Grosskurth, Saidi H. Kapiga

**Affiliations:** 1 Department of Infectious Disease Epidemiology, London School of Hygiene and Tropical Medicine, London, United Kingdom; 2 Mwanza Centre, National Institute for Medical Research, Mwanza, Tanzania; 3 Mwanza Intervention Trials Unit (MITU), Mwanza, Tanzania; University of Stellenbosch, SOUTH AFRICA

## Abstract

**Introduction:**

Alcohol use is a global public health problem, including as a risk factor for HIV infection, but few data are available on the epidemiology of alcohol use and alcohol use disorders (AUD) among young people in sub-Saharan Africa.

**Methods:**

We conducted a cross-sectional survey among 4 groups of young people aged 15–24 years old (secondary school students, college/university students, employees of local industries and casual labourers) in two regions (Kilimanjaro and Mwanza) of northern Tanzania. Using a multistage stratified random sampling strategy, we collected information on demographics, alcohol use, and behavioural factors. We screened severity of alcohol use using the Alcohol Use Disorder Identification Test (AUDIT) and estimated the quantity and frequency of alcohol consumption using the timeline-follow-back-calendar (TLFB) method.

**Results:**

A total of 1954 young people were surveyed. The prevalence of reported alcohol use was higher among males (47–70% ever users and 20–45% current users) than females (24–54% ever users and 12–47% current users). Prevalence of use was substantially higher in Kilimanjaro than Mwanza region. In both regions, participants reported high exposure to alcohol advertisements, and wide alcohol availability. College students reported the highest prevalence of current alcohol use (45% among males; 26% among females) and of heavy episodic drinking (71% among males; 27% among females) followed by casual labourers. Males were more likely to have AUD (an AUDIT score ≥8) than females, with 11–28% of males screening positive for AUD. Alcohol use was associated with male gender, being in a relationship, greater disposable income, non-Muslim religion and a higher number of sexual partners.

**Conclusions:**

Alcohol use is a significant problem among young people in northern Tanzania. There is an urgent need to develop, pilot and deliver interventions to help young people delay initiation and reduce levels of harmful drinking, particularly among college students and casual labourers.

## Introduction

Excessive alcohol use is a global public health problem accounting for about 6% of mortality and 5% of disability adjusted life year’s (DALYs) lost worldwide [1]. The World Health Organization (WHO) estimate that, globally, about 53% of people aged 15 years and above have ever used alcohol and 39% used it in the last year [1]. Within Africa, an estimated 43% of those aged 15 years or above have ever used alcohol and 30% used it in the last year [1]. The reported prevalence of alcohol use disorders (AUD) (defined by an Alcohol Use Disorders Identification Test (AUDIT) score ≥8) is estimated at 4% globally and 3% in Africa, and is generally more prevalent among men [1]. AUD are associated with acute and long-term medical complications [1–3] and may interfere with the treatment of chronic diseases such as diabetes and HIV/AIDS due to poor treatment adherence [4, 5]. Alcohol use and AUD are also associated with intentional and unintentional injuries, domestic violence, unemployment and decreased work productivity [6–10].

Data from industrialized countries show that excessive alcohol use often begins at young age [11–14]. In 2012, according to WHO, 46% of the world’s adolescents aged 15–19 years reported having ever used alcohol, and 34% had used it in the last year. In Africa, these estimates were 41% and 29% respectively[1]. The prevalence of heavy episodic drinking in adolescents was 8% globally and 6% in Africa, and higher among adolescents than adults[1]. Adolescents and young adults tend to experiment, and the intake of excessive amounts of alcohol may be a consequence of this [11–14]. Previous studies from Europe, America and some settings in sub-Saharan Africa (SSA) show that risk factors predisposing young people to excessive alcohol use include male gender, peer pressure, family history of alcohol abuse, unstable employment, economic uncertainties, poor social and coping skills, increased alcohol availability, and positive expectations regarding alcohol use [15–18]. In recent years, alcohol advertisements have become widespread in SSA and in other regions of the world. Most advertisements propagate drinking as modern and associated with occupational and sexual achievements [19–21].

Our recent systematic review showed that alcohol use is common among young people aged 15–24 years in East Africa with the highest levels recorded among sex workers and college students [22]. However, the review also highlighted a lack of data on the prevalence of alcohol use among young people in this region [22]. To inform health policy and intervention planning, we conducted this study among different groups of young people in two populous regions of northern Tanzania. We aimed to determine the prevalence of alcohol use and AUD, and describe factors associated with alcohol use and AUD in the study population.

## Methods

### Study setting

Between July 2012 and June 2013, we conducted a cross-sectional survey to determine the patterns of and risk factors for alcohol use and AUD among young people in Kilimanjaro and Mwanza regions of northern Tanzania. These regions were purposely selected to represent divergent socio-economic conditions and cultural and social norms for young people. We surveyed young men and women from four groups: (i) secondary school students, (ii) college students, (iii) young people employed in local industries and (iv) casual labourers recruited from construction sites, car workshops and farms, as a proxy for youth without employment as these are otherwise difficult to identify. We defined casual labourers as young people without formal employment contracts, mostly receiving their remunerations on daily or weekly basis. This group included mostly young people without formal training and some mechanics and artisans, however, some worked in formal sectors (such as coffee farms, sugar plantations). We obtained ethical approvals from the Tanzania National Health Research Ethics Committee (NIMR/HQ/R.8a/vol. IX/1339) and the London School of Hygiene and Tropical Medicine (LSHTM ethics ref: 6149).

### Sampling strategy

We used a stratified multistage sampling scheme, with 4 strata (2 in each region): Nyamagana and Sengerema, an urban and rural district, respectively, in Mwanza region, and Moshi urban and Moshi rural districts in Kilimanjaro. Owing to funding constraints, not all districts in each region could be sampled; therefore, the districts were randomly selected from among the rural and urban districts in each region. Study participants were aged 15–24 years, provided written informed assent or consent and lived in one of the selected districts. Primary sampling units (PSUs) were educational institutions and work places. Sampling was done without replacement. We originally planned to survey 128 men and 128 women from each of the 4 groups from each district (256 men and 256 women per group. However, there were fewer than expected young persons among employees of local industries and casual labourers, and therefore we surveyed all available individuals within the eligible age range for these two groups. In Mwanza region, we expanded recruitment for these two groups to all available individuals from the other three districts; with the exception of Ukerewe district, an island archipelago that was excluded for logistical reasons. Recruitment was not expanded in Kilimanjaro region because we reached targeted sample for the casual labourers in Moshi urban and rural districts, and there were no operational local industries in other rural districts.

Within each district, educational institutions and classes were selected by simple random sampling, using random numbers generated by Stata 12.1 (StataCorp (2011), College Station, TX).

#### Secondary school students

We obtained a list of all secondary schools in the selected districts, including government and private schools, and boarding and day schools. In Nyamagana, Sengerema, Moshi urban and Moshi rural districts, there are 44, 40, 30 and 96 secondary schools, respectively. We randomly selected two schools from each district. From each school, we randomly selected two classes (excluding final year classes preparing for the national examinations), and from each class we randomly selected 16 boys and 16 girls.

#### Students of colleges and universities

Students enrolled in colleges and universities pursuing ordinary diploma, advanced diploma or undergraduate degree programs were eligible to take part in the study. In Nyamagana, Sengerema, Moshi urban and Moshi rural districts, there were 4, 3, 3 and 5 colleges/universities respectively, of which 2 were randomly selected per district. In each institution, we obtained a list of courses/programmes and randomly selected two for inclusion in the study. From each course/programme, we randomly select 16 men and 16 women.

#### Industrial employees

We surveyed employees from all identified non-alcohol producing industries who had been employed for at least six months. From each work place, we obtained a list of eligible workers from the employers and surveyed everybody who consented. Industries comprised soft drink and mineral water-bottling factories, and fish and food processing industries (urban Mwanza districts), cotton ginneries and metal processing workshops (rural Mwanza districts); soft drink, paper, tannery and match industries (Moshi urban) and sugar cane and coffee plantations (Moshi rural).

#### Casual labourers

We surveyed all available eligible short-term workers we could find at all identified building and road construction sites, car workshops and plantations from all districts in Mwanza region except Ukerewe and Moshi urban and Moshi rural districts in Kilimanjaro region.

### Data collection and ethical considerations

After the sites were selected, we obtained permission to conduct interviews from the head of the educational institutions or companies involved. Trained research assistants provided information about the study to groups of potential participants at the selected schools, colleges and work places. Students were informed about the study, and were invited to give written assent (if they were between 15 and 18 years old); or written consent (if they were aged 18 years or above). For day schools, one week prior to data collection, an information sheet was provided to students to present to their parents. Through this information sheet, parents were informed about the study, and invited to raise any questions, objections or concerns they might have, and to contact the investigators if needed. This gave parents an opportunity for their children to opt out of the study if they wished to do so. For boarding schools, given communication limitations in Tanzania, this procedure was not possible. In this situation, we obtained verbal permission from the respective class teachers, in addition to written personal assent or consent. This study and consent procedure was approved by the Tanzania National Health Research Ethics Committee (NIMR/HQ/R.8a/vol. IX/1339) and the Ethics Committee of the London School of Hygiene and Tropical Medicine (LSHTM ethics ref: 6149). We provided information on the effects of excessive alcohol use. We could not refer young people with hazardous/harmful alcohol use for further management and support, as such services were not available in the study settings. We plan to use findings of this study to highlight the need for alcohol interventions to address hazardous/harmful alcohol use in the study settings. All data were collected anonymously, i.e. the questionnaires did not have personal identifiers, and it was therefore not possible to trace individual responses to specific students. Participants were interviewed in private using a pre-tested structured questionnaire (S1 File). Completed questionnaires were securely stored in the field and were submitted to Mwanza Intervention Trials Unit (MITU)’s data section for further processing.

### Primary study variables

We translated English versions of data collection tools, including AUDIT alcohol screening questionnaire, into Swahili and then back translated into English, and pilot tested them before commencing data collection. These tools had been previously applied in other studies in the northern Tanzania settings by our study group.

The main outcome was prevalence of reported alcohol use (ever, in the last 12 months, the last 2 months, and the last 30 days). Other outcomes included the frequency and amount of alcohol consumed (defining 10g of pure ethanol as one standard drink)[23], the prevalence of hazardous/harmful/dependent alcohol use assessed by the WHO-AUDIT questionnaire (AUDIT score of ≥8) [24].

Additional questions on alcohol use included the perceived possible adverse effects of alcohol with regards to school or work performance, use of alcohol by siblings, exposure to alcohol advertisements, alcohol availability and personal views about alcohol; the circumstances of participants’ first ever alcohol use, the type of alcoholic beverage used at the time, type currently preferred, drinking habits over the last year. We used the alcohol timeline follow-back (TLFB) calendar method to obtain detailed information on alcohol use over the past sixty days [25]. For any day in the calendar with reported alcohol use, the number of standard drinks consumed was estimated using a pictorial display of different types of drinks (S2 File). We also asked about the consumption of traditional (locally brewed) alcoholic drinks.

We also collected information on age, disposable cash in a month, marital or relationship status, region of residence, sexual risk taking under the influence of alcohol, history of sexually transmitted infections (STIs), casual sex in the last month, and number of lifetime sexual partners and partners during the last year.

### Sample size

Due to the heterogeneity of different groups regarding socio-economic status and alcohol use, the sample size was estimated to provide adequate precision within each group. Based on the literature on alcohol use among young people in Tanzania [26–28], we assumed the overall prevalence of current alcohol use to be about 20% and chose a sample size to estimate this prevalence with a precision of 5% within each of the four groups. Assuming a design effect of 2 due to the clustered study design, the estimated minimum sample size required per group was 490 [29]. To allow for a non-response rate of about 5%, we aimed to recruit 512 participants from each group. For the investigation of risk factors associated with alcohol use in each group, this sample size provides 80% power to estimate odds ratios of 2.0 if the proportion of young people with the outcome was 15% among those unexposed.

### Data management and analysis

Field supervisors checked the completed questionnaires for consistency and quality at the end of each day. Data were double-entered using OpenClinica version 3.0.1 (OpenClinica, LLC (2014) and checked for completeness and accuracy, and were analysed using Stata 12.1 (StataCorp (2011), College Station, TX) stratified by study group. We accounted for the stratified and multi-stage survey design using STATA’s survey procedures. To allow for the differential probability of selection (since the sampling scheme was not self-weighting), we applied sampling weights for the analysis of data from the secondary school and college/universities. For employees and casual labourers, we did not apply sampling weights since we surveyed all available individuals at every eligible employment site. Due to the small number of PSUs in each stratum, we estimated variances using a repeated half-sample bootstrap algorithm that gives a less biased estimate of the variances in a situation with few PSUs, and constructed the confidence intervals using the percentiles of the bootstrap distribution[30].

For AUDIT questions, we computed total scores and categorised AUDIT scores as binary with a cutpoint of ≥8 to indicate AUD (hazardous or harmful or problematic alcohol use/possible dependence) [24]. For the amount of alcohol reported for each specific event in the TLFB calendar, we computed the number of drinking events and the consumption per event, and estimated the median and interquartile ranges.

To assess factors associated with each binary outcome (ever use, use in the last year, and hazardous alcohol use) we used logistic regression, allowing for the survey design and with sampling weights applied (except for the two work place groups). Associations with AUD were only analysed for male participants, as the number of individuals with this outcome was small among females. For the multivariable models, region (Mwanza or Kilimanjaro), religion and sex were included as a priori confounders. Other factors associated in the univariable analysis were included as they had p-values of ≤0.1. We retained these factors in the final model if they were independently associated with the outcome (p≤0.05). We report crude and adjusted odds ratios (AOR) and their 95% confidence intervals (CI). We assessed the interaction between sex and all exposure variables, and location and all exposure variables, respectively.

## Results

### Recruitment and sample characteristic

We surveyed 1954 young people, 960 (49%) in Mwanza region and 994 (51%) in Kilimanjaro region. These included 517 secondary students, 525 college and university students, 423 employees of local industries and 489 casual labourers. Response rates were high in all four groups (S1 Fig). All selected schools/workplaces consented to participate in the survey.

More men than women were recruited from local industries (71%) and from casual labourers (91%) as they are predominantly male occupation in the study settings. Demographic and behavioural characteristics are shown in Table 1, and varied between the groups by age, marital status, education level, income and sexual behaviours.

**Table 1:** General characteristics of the study populations among young people in northern Tanzania.

		Secondary school students		College and university students		Employed in local industries		Casual labourers	
Characteristic	Responses	Female	Male	Female	Male	Female	Male	Female	Male
		N (%)^1^ ^,^ ^2^	N (%)^1^ ^,^ ^2^	N (%)^1^ ^,^ ^2^	N (%)^1^ ^,^ ^2^	N (%)^1^ ^,^ ^3^	N (%)^1^ ^,^ ^3^	N (%)^1^ ^,^ ^3^	N (%)^1^ ^,^ ^3^
Total sample	N	261	256	263	262	123	300	43	446
Location	Rural	130(78.9)	132(82.1)	130(31.7)	132(37.0)	77(62.6)	134(44.7)	37(86.1)	195(43.7)
	Urban	131(21.1)	124(17.9)	133(68.3)	130(63.0)	46(37.4)	166(55.3)	6(14.0)	251(56.3)
Region	Mwanza	128(49.4)	128(42.7)	131(69.2)	130(60.7)	46(37.4)	159(53.0)	7(16.3)	231(51.8)
	Kilimanjaro	133(50.6)	128(57.3)	132(30.8)	132(39.3)	77(62.6)	141(47.0)	36(83.7)	215(48.2)
Age (years)	15–17	185(81.2)	155(74.4)	0 (0)	0 (0)	1(0.8)	6(2.0)	8(18.6)	68(15.3)
	18–20	72(17.5)	79(21.7)	30(4.8)	19(2.5)	38(30.9)	68(22.7)	16(37.2)	169(37.9)
	21–24	4(1.4)	22(3.9)	233(95.2)	243(97.5)	84(68.3)	226(75.3)	19(44.2)	209(46.9)
Religion	Muslim	52(12.2)	42(9.3)	33(10.2)	30(17.2)	23(18.7)	61(20.6)	5(11.6)	117(26.2)
	Catholic	94(39.7)	95(45.1)	102(42.4)	115(44.4)	64(52.0)	132(44.6)	26(60.5)	207(46.4)
	Protestant	115(48.1)	119(45.6)	128(47.3)	117(38.4)	36(29.3)	103(34.8)	12(27.9)	118(26.5)
	Other	0(0.0)	0(0.0)	0(0.0)	0(0.0)	0(0.0)	4(1.3)	0(0.0)	4(0.9)
Education	Primary	196(88.0)	190(89.9)	0.0	0.0	61(49.6)	152(50.7)	31(72.1)	338(75.8)
	Secondary and above	65(12.1)	66(10.1)	263(100.0)	262(100.0)	62(50.4)	148(49.3)	12(27.9)	108(24.2)
Marital status	Single	215(84.5)	217(85.7)	69(32.4)	141(57.0)	35(28.5)	148(49.3)	21(48.8)	281(63.0)
	In a relationship	46(15.5)	39(14.3)	194(67.6)	121(43.0)	88(71.5)	152(50.7)	22(51.2)	165(37.0)
Disposable cash in a month	Median in USD and IQR	6.25[3.13–18.75]	9.38[3.13–15.63]	125[62.5–125]	125[59.38–156.25]	53.13[46.88–62.5]	56.25[43.75–75.00]	25.00[12.5–37.5]	37.5[18.75–62.50]
Ever had sex	Yes	27(9.4)	107(40.1)	169(66.3)	227(89.0)	103(83.7)	271(90.3)	39(90.7)	361(80.9)
Sexual intercourse last year	Yes	20(6.2)	61(20.0)	154(59.3)	195(79.3)	98(79.7)	230(76.7)	36(83.7)	297(66.6)
Number of life time sexual partners	0–1	260(99.8)	214(84.5)	205(74.9)	101(29.7)	80(65.5)	92(30.7)	20(46.5)	172(38.6)
	2 and more	1(0.2)	42(15.5)	58(25.1)	161(70.3)	43(35.0)	208(69.3)	23(53.5)	274(61.4)
Sexual partners in the last year	0–1	259(99.2)	243(95.4)	250(91.6)	206(72.0)	117(95.9)	232(77.3)	40(93.0)	307(68.8)
	2 and more	2(0.8)	12(4.6)	13(8.4)	56(28.0)	6(4.1)	68(22.7)	3(7.0)	139(31.2)
Casual sex encounter last month	None	260(99.4)	245(96.1)	259(97.7)	226(86.9)	118(95.9)	266(88.7)	40(93.0)	382(85.7)
	one and more	1(0.6)	9(3.9)	4(2.3)	36(13.1)	5(4.1)	34(11.3)	3(7.0)	64(14.3)
Condom use	Never	9(4.2)	46(19.5)	22(8.1)	18(4.5)	33(26.8)	48(16.0)	10(23.3)	91(20.4)
	sometimes	8(2.2)	17(8.5)	49(21.0)	53(31.1)	33(26.8)	74(24.7)	20(46.5)	109(24.4)
	Most of the time	10(3.0)	44(12.1)	98(37.2)	156(53.4)	37(30.1)	149(49.7)	9(20.9)	161(36.1)
	Never had sex	234(90.6)	149(59.9)	94(33.7)	35(11.0)	20(16.3)	29(9.7)	4(9.3)	85(19.1)

^1^Actual number of respondents, without sampling weights applied.

^2^Proportions are weighted estimates.

^3^Proportions without sampling weights applied.

Most participants reported having seen alcohol advertisements in the last month (from 67% among school students to 89–99% among other groups), many reported having seen alcohol advertisements almost daily (37–79%) and the majority in each group reported having seen movie or cinema actors drinking alcohol in most of the films (61–92%). About two-thirds (64%) of secondary school students and almost all (>95%) of participants from other groups reported that it was very easy to obtain alcohol if they wanted. Almost all participants perceived alcohol as harmful (95–100%) and about half reported having siblings who drank alcohol (30–59%) (Table 2).

**Table 2:** Description of potential factors related to alcohol use among young people in northern Tanzania.

		Secondary school students		College and university students		Employed in local industries		Casual labourers	
Factor	Responses	Female	Male	Female	Male	Female	Male	Female	Male
		N (%)^1^ ^,^ ^2^	N (%)^1^ ^,^ ^2^	N (%)^1^ ^,^ ^2^	N (%)^1^ ^,^ ^2^	N (%)^1^ ^,^ ^3^	N (%)^1^ ^,^ ^3^	N (%)^1^ ^,^ ^3^	N (%)^1^ ^,^ ^3^
Total sample	N	261	256	263	262	123	300	43	446
Saw alcohol adverts in last 30 days among everyone	Never	60(31.7)	44(32.8)	5(1.0)	7(0.8)	13(10.6)	9(3.0)	1(2.3)	5(1.1)
	Sometimes	79(31.0)	74(29.6)	66(20.0)	51(20.2)	26(21.1)	90(30.0)	10(23.3)	90(20.2)
	Almost daily	122(37.4)	138(37.6)	192(79.0)	204(79.0)	84(68.3)	201(67.0)	32(74.4)	351(78.7)
Adverts in sports, social gathering and community events	Do not go	69(32.5)	48(18.0)	41(10.1)	24(5.5)	38(30.9)	58(19.3)	8(18.6)	95(21.3)
	sometimes	92(36.9)	94(40.9)	67(24.1)	82(35.4)	19(15.5)	106(35.3)	11(25.6)	158(35.4)
	Most of the time	100(30.6)	114(41.1)	155(65.8)	156(59.1)	66(53.7)	136(45.3)	24(55.8)	193(43.3)
Saw actors drinking alcohol in movies among everyone	Do not watch TV	24(12.9)	16(7.0)	4(1.4)	6(3.3)	22(17.9)	25(8.3)	8(18.6)	37(8.3)
	Sometimes	57(25.8)	57(25.5)	24(7.1)	45(19.5)	20(16.3)	81(27.0)	8(18.6)	111(24.9)
	Most of the time	180(61.3)	183(67.5)	235(91.5)	211(77.2)	81(65.9)	194(64.7)	27(62.8)	297(66.7)
Ease of obtaining alcohol	Difficult	64(35.6)	53(35.8)	2(1.3)	5(1.1)	3(2.5)	10(3.3)	2(4.7)	12(2.7)
	Easy	197(64.4)	203(64.2)	260(98.7)	257(98.9)	119(97.5)	290(96.7)	41(95.4)	434(97.3)
Taught in school about problems associated with alcohol	Yes	206(76.0)	211(78.8)	227(87.1)	230(86.4)	90(73.2)	234(78.0)	30(69.8)	307(68.8)
Brothers and sisters who drink alcohol	Yes	80(31.5)	81(30.3)	130(59.0)	130(48.6)	44(36.7)	100(33.9)	22(52.4)	183(41.6)
Perceive drinking alcohol may be harmful	Yes	260(99.2)	252(98.6)	262(99.8)	262(100.0)	121(98.4)	294(98.0)	42(97.7)	424(95.1)

^1^Actual number of respondents, without sampling weights applied.

^2^Proportions are weighted estimates.

^3^Proportions without sampling weights applied.

### Initiation and persistence of alcohol use

The majority of participants reported that they had their first drink at a social event (during a public holiday, a family celebration, wedding (S1 Table). The first drink was most commonly bottled beer (31%-66%). Local brew was also a common first drink (36%-45%), especially among secondary school girls. The main motive reported for initiating alcohol use was “wanting to try” or a combination of reasons for example “wanting to try and convinced by a friend”. Among non-drinkers, reasons for avoiding alcohol included the influence of parents or other relatives, religion and being afraid of side effects. Among previous users of alcohol, the main reason for not drinking was a dislike of alcohol (67% of the female casual workers to 99% of the male college students who had been abstinent in the last year).

### Prevalence and epidemiology of reported ever and recent use of alcohol

Reported alcohol use was common and was generally higher among males than females across all study groups (Table 3). However, the differences were significant only among secondary and college/university students (p<0.001) which were the only groups where numbers of females permitted a formal comparison by gender. Prevalence of ever use of alcohol was highest amongst male college students (70.4%, 95%CI: 54.8–74.2) and male casual labourers (61.0%, 95% CI: 51.4–67.7), and lowest among female secondary students (24.0%, 95%CI: 19.2–30.6). The prevalence of recent alcohol use (reported use in the last two months) followed a similar distribution pattern, although among casual labourers it was higher among women than men, and ranged from 3% among female school students to 30% among female casual labourers. Reported alcohol use was significantly higher in Kilimanjaro than Mwanza region for all groups except for college students.

**Table 3:** Prevalence of alcohol use and alcohol use disorders (AUD) among young people in northern Tanzania.

		Secondary school students^2^		College and university students^2^		Employed in local industries^3^		Casual labourers^3^	
Characteristic	Responses	Female	Male	Female	Male	Female	Male	Female	Male
Total sample	N^4^	261	256	263	262	123	300	43	446
Ever used alcohol	Total	24.0[19.2–30.6]	47.0[37.9–55.3]	46.6[29.3–52.2]	70.4[54.8–74.2]	47.2[37.7–55.7]	50.7[39.9–63.9]	53.5[7.7–75.0]	61.0[51.4–67.7]
	Kilimanjaro	34.7[28.5–40.8]	63.9[59.0–66.1]	40.5[26.8–52.1]	67.0[50.4–74.0]	57.1[53.3–68.3]	68.1[55.3–81.8]	52.8[0.0–59.4]	70.7[60.0–76.8]
	Mwanza	12.9[8.5–16.0]	24.2[22.3–25.8]	49.3[32.7–52.2]	72.7[61.4–74.3]	30.4[18.2–50.0]	35.2[26.2–48.9]	57.1[0.0–100]	51.9[45.0–58.6]
Alcohol use in the last year	Total	11.7[5.8–17.9]	24.0[15.6–30.5]	25.5[14.4–29.1]	45.2[28.6–49.1]	24.4[15.0–34.4]	20.3[14.2–28.2]	46.5[0.0–56.7]	29.1[22.7–34.2]
	Kilimanjaro	16.6[10.4–22.6]	35.5[31.4–37.3]	21.8[12.5–29.6]	41.0[23.2–48.5]	32.5[26.3–50.0]	33.3[23.6–45.0]	50.0[0.0–56.3]	35.3[27.6–40.6]
	Mwanza	6.6[0.0–11.1]	8.6[4.7–14.4]	27.2[16.9–29.0]	48.0[37.3–49.5]	10.9[0.0–30.8]	8.8[3.8–17.5]	28.6[0.0–66.7]	23.4[16.9–29.4]
Alcohol use in the last two months	Total	2.8[1.1–5.3]	11.6[4.7–17.0]	16.5[7.9–19.2]	26.8[20.2–39.1]	11.4[6.3–18.3]	13.7[8.5–21.2]	30.2[0.0–36.6]	18.2[11.7–22.7]
	Kilimanjaro	5.2[2.5–7.8]	19.0[11.5–22.3]	13.7[6.3–19.9]	33.6[17.3–40.6]	16.9[10.7–30.0]	23.4[14.1–35.1]	33.3[0.0–37.5]	24.7[17.8–29.6]
	Mwanza	0.4[0.0–1.3]	1.8[0.0–4.6]	17.8[10.6–19.0]	22.5[20.8–33.8]	0	5.0[0.0–10.7]	14.3[0.0–28.6]	12.1[7.2–16.8]
Alcohol use in the last month	Total	0.9[0.1–2.0]	6.6[4.7–8.0]	13.3[7.4–15.1]	25.3[19.5–35.9]	7.3[3.1–12.9]	12.7[7.9–19.1]	27.9[0.0–33.8]	16.1[9.9–21.0]
	Kilimanjaro	1.4[0.2–2.5]	10.5[10.0–11.5]	10.1[6.0–13.4]	31.0[14.4–38.0]	11.7[5.6–25.0]	21.3[12.8–32.3]	30.6[0.0–34.4]	22.8[15.6–28.0]
	Mwanza	0.4[0.0–1.3]	1.3[0.0–3.4]	14.7[9.6–15.6]	21.6[20.7–28.0]	0	5.0[0.0–10.7]	14.3[0.0–28.6]	10.0[4.6–14.6]
Alcohol use disorders (AUD)^1^	Total	0.3[0.0–0.7]	10.6[3.8–16.6]	6.5[2.3–9.8]	27.5[13.6–30.9]	1.6[0.0–3.3]	6.0[2.8–10.7]	7.0[0.0–9.1]	13.7[8.0–18.3]
	Kilimanjaro	0.1[0.0–0.3]	18.4[10.3–22.1]	6.3[0.0–11.6]	20.9[8.0–26.7]	2.6[0.0–6.7]	8.5[4.5–12.9]	8.3[0.0–9.4]	14.9[6.7–20.8]
	Mwanza	0.4[0.0–1.3]	0	6.5[5.6–6.7]	31.7[22.5–33.0]	0	3.8[0.0–12.5]	0	12.6[7.6–17.0]

^1^AUD defined as AUDIT score ≥8.

^2^Prevalences and confidence intervals are weighted estimates, adjusted for survey design with sampling weights applied.

^3^Standard errors are adjusted for the survey design but without sampling weights applied.

^4^Actual number of respondents, without sampling weights applied.

Ever use of alcohol was strongly associated with residence in Kilimanjaro among secondary students (AOR = 4.36, 95%CI: 2.71–9.21), employees in local industries (AOR = 5.28, 95%CI: 2.62–11.61), and casual labourers (AOR = 3.53, 95% CI: 1.73–5.58) (Table 4). Other factors independently associated with ever use of alcohol included male gender (AORs ranging between 1.25 to 4.00), having siblings who drink alcohol, being in a relationship, and among college students having disposable cash that was above average. Belonging to a non-Muslim faith was generally associated with ever use of alcohol. Associations with reported alcohol use in the last year followed a similar pattern.

**Table 4:** Factors associated with reported ever use of alcohol among young people in northern Tanzania.

		Secondary school students			College and university students			Employed in local industries			Casual labourers		
Variables	Categories	Number reporting ever use (n = 164)	Crude estimates	Adjusted estimates	Number reporting ever use (n = 280)	Crude estimates	Adjusted estimates	Number reporting ever use (n = 210)	Crude estimates	Adjusted estimates	Number reporting ever use(n = 295)	Crude estimates	Adjusted estimates
			OR (95%CI)	AOR^1^ (95%CI)		OR (95%CI)	AOR^2^ (95%CI)		OR (95%CI)	AOR^3^ (95%CI)		OR (95%CI)	AOR^4^ (95%CI)
Area	Rural	78(29.8)	1	1	120(45.8)	1	1	118(52.9)	1	1	168(64.4)	1	1
	Urban	86(33.7)	0.83[0.46–1.68]	1.70[1.06–5.22]	160(60.8)	1.54[0.65–3.25]	1.95[0.92–2.79]	92(46.0)	0.76[0.43–2.11]	0.85[0.51–2.31]	127(55.7)	0.70[0.44–1.66]	0.67[0.42–1.53]
Region	Mwanza	53(20.7)	1	1	130(49.8)	1	1	70(34.2)	1	1	124(52.1)	1	1
	Kilimanjaro	111(42.5)	4.61[3.07–6.37]	4.36[2.71–9.21]	150(56.8)	0.82[0.36–2.31]	0.77[0.49–1.89]	140(64.2)	3.46[1.75–6.17]	5.28[2.62–11.61]	171(68.1)	1.97[0.99–3.01]	3.53[1.73–5.58]
Sex	Female	63(24.1)	1	1	111(42.2)	1	1	58(47.2)	1	1	23(53.5)	1	1
	Male	101(39.5)	2.81[2.12–3.36]	3.18[2.64–4.66]	169(64.5)	2.73[2.60–3.09]	4.00[2.39–5.17]	152(50.7)	1.15[0.75–2.08]	1.25[0.66–2.62]	272(61.0)	1.36[0.56–12.27]	2.67[0.28–30.27]
Age	15–19	136(30.0)	1	1	5(41.7)	1	1	22(37.9)	1	1	98(50.3)	1	1
	20–24	28(43.8)	1.73[1.11–2.63]	1.06[0.75–2.03]	275(53.6)	1.31[0.44–1.33]	0.64[0.26–0.64]	188(51.5)	1.74[1.12–3.89]	1.66[0.98–3.26]	197(67.0)	2.01[1.34–2.93]	1.59[0.78–2.46]
Marital status	Single	116(24.1)	1	1	100(47.6)	1	1	76(41.5)	1	1	169(56.0)	1	1
	In relationship	48(56.5)	4.72[2.91–6.27]	4.29[2.35–8.88]	180(57.1)	2.06[1.20–2.58]	2.65[1.12–3.84]	134(51.9)	1.78[1.12–2.91]	1.84[1.07–3.66]	126(67.4)	1.63[1.33–2.16]	1.26[0.93–1.84]
Disposable cash in a month	Below median for the group	69(24.7)	1	1	131(43.1)	1	1	100(47.4)	1	1	140(56.2)	1	1
	Above median for the group	95(39.9)	2.80[1.18–4.56]	1.78[1.31–5.12]	149(67.4)	2.28[1.31–9.20]	2.14[1.00–10.82]	110(51.9)	1.2[0.78–2.04]	0.88[0.40–1.61]	155(64.6)	1.42[1.11–2.06]	1.24[0.73–2.13]
Siblings drink alcohol	No	76(21.4)	1	1	106(40.0)	1	1	125(44.8)	1	1	145(51.1)	1	1
	Yes	88(54.7)	4.31[3.46–5.30]	3.95[2.74–7.31]	174(66.9)	2.26[1.67–4.63]	1.81[1.16–4.29]	85(59.0)	1.77[1.15–2.81]	1.54[0.83–2.81]	150(73.2)	2.61[1.53–3.60]	1.87[0.95–2.75]
Religion	Muslim	18(19.5)	1	1	21(33.3)	1	1	38(45.2)	1	1	60(49.2)	1	1
	Catholic	74(39.2)	2.56[0.74–4.92]	2.73[1.09–4.99]	135(62.2)	4.61[1.76–7.08]	6.36[1.39–12.50]	110(56.1)	1.55[0.93–2.44]	2.02[1.20–4.04]	165(70.8)	2.51[1.36–4.31]	2.58[1.56–5.00]
	Protestants	72(30.8)	1.79[1.00–2.85]	2.06[0.83–3.1]	124(50.6)	2.50[0.70–4.61]	3.25[0.63–7.09]	61(43.9)	0.95[0.59–1.40]	1.01[0.53–1.66]	68(52.3)	1.13[0.57–1.96]	1.08[0.45–2.07]
Life time sexual partners	0–1	135(28.5)	1	1	123(40.2)	1	1	71(41.3)	1	1	86(44.8)	1	1
	2 and more	29(67.4)	6.46[3.93–15.08]	2.21[1.15–9.67]	157(71.7)	3.49[3.17–4.11]	1.68[1.27–3.43]	139(55.4)	1.77[1.14–3.63]	2.69[1.65–5.49]	209(70.4)	2.93[2.25–4.38]	3.10[2.15–5.40]

All OR and AOR are adjusted for the survey design.

AOR^1^- adjusted for location, sex, age, marital status, income, having siblings who drink alcohol, religion and number of lifetime partners.

AOR^2^- adjusted for location, sex, marital status, disposable cash, having siblings who drink alcohol, religion and number of life time partners.

AOR^3^- adjusted for location, sex, age, marital status, disposable cash, having siblings who drink alcohol, religion and number of lifetime partners.

AOR^4^ -adjusted for location, sex, age, marital status, disposable cash, having siblings who drink alcohol, religion and number of life time partners.

Ever use of alcohol and use in the last year was associated with reporting two or more sexual partners both over lifetime and last year across all groups of young people (Table 4), with AORs ranging from 1.2–9.0.

### Prevalence and epidemiology of AUD

AUD was highly prevalent among male college students (27.5%, 95% CI: 13.6–30.9%); and common among male casual labourers (13.7%, 95%CI: 8.0–18.3%) and male secondary students (10.6%, 95%CI: 3.8–16.6%) (Table 3). Relatively few women screened positive for AUD, and this was highest among casual labourers (7.0%, 95% CI: 0.0–9.1%) and college students (6.5%, 95%CI 2.3–9.8%). The prevalence of AUD did not vary significantly between regions except for male secondary school students from Kilimanjaro region who were more likely to be affected than male secondary school students in Mwanza. AUD was associated with older age, higher disposable cash in a month, having a sibling who drank alcohol, and having ≥2 lifetime sexual partners (Table 5).

**Table 5:** Factors associated with Alcohol Use Disorders (AUDIT score ≥8) among young males in northern Tanzania.

		Secondary school students^3^		College and university students			Employed in local industries^3^		Casual labourers		
Variables	Categories	Number screened positive for AUD (n = 15)	Crude estimates	Number screened positive for AUD (n = 53)	Crude estimates	Adjusted estimates	Number screened for AUD (n = 18)	Crude estimates	Number screened for AUD (n = 61)	Crude estimates	Adjusted estimates
			OR (95%CI)		OR (95%CI)	AOR^1^ (95%CI)		OR (95%CI)		OR (95%CI)	AOR^2^ (95%CI)
Area	Rural	12(9.1)	1	18(13.6)	1	1	14(10.0)	1	40(17.9)	1	1
	Urban	3(2.4)	0.1[0.10–0.69]	35(26.9)	1.85[0.89–7.99]	1.33[0.75–4.71]	4(2.5)	0.23[0.08–1.01]	21(9.4)	0.48[0.26–1.53]	0.47[0.21–1.62]
Location	Mwanza	0(0.4)		29(22.3)	1	1	6(3.8)	1	29(12.6)	1	1
	Kilimanjaro	15(11.7)		24(18.2)	0.57[0.18–1.25]	0.51[0.17–1.11]	12(8.5)	2.37[0.51–16.73]	32(14.9)	1.22[0.39–2.59]	1.91[0.53–4.67]
Marital status	Single	8(3.7)	1	20(14.2)	1	1	11(7.4)	1	25(8.9)	1	1
	In relationship	7(18.0)	9.78[4.15–14.35]	33(27.3)	1.78[1.16–2.00]	1.42[0.78–1.48]	7(4.6)	0.60[0.26–1.55]	36(21.8)	2.86[1.76–7.13]	1.70[0.87–4.27]
Disposable cash in a month	Below median for the group	1(0.7)	1	21(13.8)	1	1	8(5.4)	1	17(7.7)	1	1
	Above median for the group	14(12.4)	19.85[4.33–8.09]	32(29.1)	0.90[0.35–8.41]	0.66[0.21–7.68]	10(6.5)	1.22[0.32–5.73]	44(19.6)	2.95[1.73–14.50]	2.18[1.19–17.91]
Brothers and sisters drink alcohol	No	7(4.0)	1	17(12.9)	1	1	9(4.5)	1	24(9.1)	1	1
	Yes	8(9.9)	2.56[1.88–7.77]	36(27.7)	2.59[2.29–4.34]	2.50[2.33–4.28]	9(9.0)	2.10[0.77–4.01]	37(20.2)	2.52[1.20–4.31]	2.14[1.00–4.62]
Sexual partners last year	0–1	9(3.7)	1	31(15.1)	1	1	12(5.2)	1	22(7.2)	1	1
	2 and more	5(41.7)	37.1[21.13–225.47]	22(39.3)	2.17[1.07–4.04]	1.46[0.77–2.72]	6(8.8)	1.77[0.59–3.56]	39(28.1)	5.05[2.98–17.36]	3.97[2.33–16.63]

All OR and AOR are adjusted for the survey design.

AOR^1^-adjusted for location, marital status, having siblings who drink alcohol and number of sexual partners in the last year.

AOR^2^-adjusted for location, age, marital status, having siblings who drink alcohol and number of sexual partners in the last year.

^3^ No AORs for secondary school students and local industry employees due to small numbers of individuals who screened positive for AUD

### Patterns of alcohol use in the last year

Among participants who had used alcohol during the last year, most had a preference for bottled beer (from 24% of male secondary students to 60% of male college students), and wine was also popular among females (Table 6). The consumption of locally brewed drinks was reported by up to 35% of participants, although not among college students (Table 6). Male college students and casual workers reported the highest frequency of heavy episodic drinking (Table 6). For example, 64% of male college students reported to have more than 6 drinks on a typical drinking occasion, and 11–14% of college students and casual labourers reported having such occasions every week.

**Table 6:** Patterns of alcohol use among young people reported alcohol use in the last year in northern Tanzania.

		Secondary school students^2^		College students^2^		Local industries employees^3^		Casual labourers^3^	
Variables	Responses	Female	Male	Female	Male	Female	Male	Female	Male
Sample	N^1^	33	48	64	100	30	61	20	130
All who had a drink in the last year	Median days drinking in a week	0[0–0]	1[1–2]	2[1–2]	2[1–2]	1[1–1]	1[1–2]	2[1–3]	2[1–2]
	Drinks per day-median	2.0[1.0–6.0]	4.0[3.0–8.0]	6.0[2.0–6.0]	9.0[6.0–12.0]	2.0[1.0–4.0]	6.0[3.0–9.0]	3.0[1.0–4.0]	6.0[2.0–9.0]
Usual drink among last year drinkers	Bottled beer	32.4[0.0–48.5]	23.7[17.3–27.0]	46.8[40.2–88.5]	60.1[20.1–83.9]	43.3[26.1–66.7]	47.5[28.0–59.7]	40.0[0.0–100]	43.9[29.5–60.8]
	Wine	22.8[11.8–41.6]	0.9[0.0–2.6]	25.3[1.6–31.8]	1.1[0.2–5.9]	6.7[0.0–23.5]	0	0	0
	Spirit/liquor	9.1[1.0–17.3]	26.3[22.0–28.2]	0.9[0.0–5.8]	3.0[0.0–8.0]	0	11.5[2.3–22.5]	0	10.8[6.2–15.1]
	Local beer/spirit/liquor	16.7[0.7–69.7]	24.9[16.3–46.9]	0	0.6[0.0–4.9]	33.3[0.0–54.8]	13.1[3.4–22.6]	35.0[0.0–38.9]	13.1[2.5–19.6]
	Other	1.8[0.0–4.9]	0	1.2[0.6–5.2]	0	0	0	0	0
	more than one type	17.3[0.0–32.2]	24.3[8.2–32.4]	25.8[1.9–39.7]	35.1[14.7–69.9]	16.7[6.1–25.0]	27.9[18.2–44.2]	25.0[0.0–27.8]	32.3[22.7–40.1]
Drink alcohol	Monthly or less	96.4[90.2–100]	82.1[77.9–90.9]	69.4[66.3–86.7]	55.2[49.3–64.8]	93.3[84.6–100]	78.7[69.5–89.8]	75.0[0.0–100.0]	66.9[57.2–81.4]
	2–4 times a month	3.6[0.0–9.8]	13.8[9.1–18.1]	17.1[4.5–21.2]	31.9[23.9–36.4]	3.3[0.0–12.5]	16.4[5.7–25.7]	15.0[0.0–16.7]	23.1[8.9–33.3]
	2 and more times a week	0	4.1[0.0–6.3]	13.5[5.6–24.8]	13.0[9.8–14.3]	3.3[0.0–8.2]	4.9[0.0–11.4]	10.0[0.0–11.1]	10.0[4.2–13.0]
Standard drinks in a typical day	1 or 2	56.4[34.8–97.8]	25.6[12.7–57.4]	32.3[7.9–40.2]	6.2[1.4–14.1]	53.3[21.6–76.5]	19.7[10.0–30.4]	45.0[0.0–50.0]	27.1[18.1–34.9]
	3 or 4	15.4[0.0–24.3]	31.3[8.0–40.9]	9.3[2.9–44.7]	13.7[11.7–26.0]	26.7[3.6–50.9]	32.8[21.1–47.9]	30.0[0.0–100.0]	16.3[5.7–24.7]
	5 or 6	26.0[0.0–48.2]	6.4[0.0–8.9]	37.2[18.0–43.9]	16.0[9.7–18.6]	13.3[0.0–23.5]	23.0[10.8–34.4]	10.0[0.0–11.1]	12.4[7.7–18.4]
	Above 6^4^	2.3[0.0–6.2]	36.7[26.8–41.4]	21.2[9.7–51.7]	64.1[47.8–69.2]	6.7[0.0–25.0]	24.6[12.3–35.1]	15.0[0.0–100.0]	44.2[38.5–52.4]
Occasions with six or more drinks	Never	58.8[50.1–72.3]	36.9[24.4–61.4]	31.2[16.6–38.6]	8.7[2.5–20.9]	56.7[28.6–76.5]	34.4[21.6–50.0]	60.0[0.0–100.0]	39.5[30.0–48.0]
	Less than monthly	31.9[24.4–49.6]	52.0[30.3–63.0]	47.1[42.9–59.3]	54.1[38.8–56.5]	40.0[23.4–69.2]	50.8[32.2–68.6]	35.0[0.0–100.0]	38.0[28.3–53.3]
	Monthly	7.5[0.0–16.1]	4.3[0.0–6.4]	10.4[0.0–12.2]	25.6[22.7–27.0]	0	4.9[0.0–10.7]	0	8.5[0.0–16.2]
	Weekly	1.8[0.0–4.9]	6.8[5.6–9.9]	11.3[5.6–14.9]	11.6[5.8–16.1]	3.3[0.0–8.2]	9.8[2.1–16.0]	5.0[0.0–5.6]	14.0[7.8–17.7]

^1^Actual number of respondents without sampling weights applied.

^2^Weighted estimates.

^3^Estimates without sampling weights applied.

^4^Heavy episodic drinking is defined as average of 6 or more standard drinks in a drinking occasion.

### Frequency and amount of alcohol used over the last 2 months

Alcohol use in the last 2 months was reported by 3% of female and 9% of male secondary school students; 16% of female and 27% of male college students; 11% of female and 14% of male employees; and by 30% of female and 18% of male casual workers respectively (S2 Table). Based on the TLFB method, male college students and male casual labourers reported the highest alcohol consumption (71% and 57% respectively) (S2 Table). Male employees and female casual labourers reported a similar number of drinking events with a lower median monthly number of standard drinks. Women of the other three sub-groups reported low levels of alcohol consumption.

## Discussion

Alcohol use was common among young people in northern Tanzania. Across different groups the prevalence was 47–70% for ever use and 20–45% for use during the past year, and was particularly high among college students and casual workers; and with the exception of female casual workers higher among men than women. Estimated alcohol use in our study was greater than the WHO estimates for Africa among young people and than data collated in a systematic review of alcohol use among young people in East Africa [1, 22]. Our findings were similar to the estimated prevalence of alcohol use among young people in the United States (US) (71% for ever use, 53% for current use) but lower than estimates from Europe[1].

We found the prevalence of episodic drinking to be similar to that reported for the general population and for college students in the US and Europe [1, 31]. Young men were more likely to screen positive for hazardous/harmful/dependent alcohol use based on AUDIT and this was consistent with other reports from Africa and elsewhere in the world [1, 32].

Reported alcohol use was higher in Kilimanjaro than in Mwanza region, possibly due to local cultural beliefs in Kilimanjaro that encourage alcohol use [33]. The role of cultural influences on the drinking behaviour of young people has been shown also by others [34]. The lack of association between alcohol use and region among college students supports this explanation, as college students involved in this survey were drawn from different cultural settings in Tanzania. Young people initiate alcohol use for a variety of reasons including a desire to better cope with stressful situations, social motives and positive enhancements experienced through alcohol [15, 35]. In this study, young people reported “wanting to try alcohol” as their main reason to initiate alcohol use. Industrially -made beer was the most commonly reported beverage. However, a substantial number of young people also reported the use of local beer, and spirits or liquors.

Socio-demographic characteristics associated with alcohol use in this study were similar to those found in studies conducted in North America and Europe included higher than average disposable cash in a month, religion (Catholic), being married or in a relationship, and having siblings who drink alcohol [34, 36, 37].

Apart from the cultural and family related influences described, a number of other structural factors may play a role in alcohol initiation and persistence among young people. Most participants reported that they had been exposed to alcohol advertisements frequently and that it was easy for them to obtain alcohol if they wanted to. Alcohol was comparatively inexpensive and affordable even to individuals who had no reliable cash income. The influence of these and possibly other structural factors seems to be substantial, as drinking habits were formed in spite of information about the negative implications of alcohol that the great majority of our participants had obtained at school, and in spite of the almost ubiquitous conviction that alcohol may be harmful.

Alcohol use was associated with risky sexual behaviours (reporting more than one sexual partner over time) in this study, in line with the scientific literature which supports associations of alcohol use, sexually transmitted infections and risky sexual behaviours indicating the need to incorporate alcohol reduction interventions in ongoing HIV interventions [3, 32, 38].

A strength of our study lies in the fact that four very different social groups were investigated, including casual workers as a proxy for the high number of unemployed youth in Tanzania. Generalisability of the results was also enhanced through the study design that either ensured a representative selection of participants within some groups or systematically enrolled all available members of other groups. Our results are generalisable for two populous regions of northern Tanzania. Given the similarity of findings between these regions, except for the generally higher alcohol intake in Kilimanjaro region, our findings provide an indication of what may be happening in other regions of Tanzania. A further strength of the study was the combination of different research tools to assess alcohol related variables, i.e. structured interview questions, AUDIT and the TLFB calendar method. This combination allowed us to triangulate results. It was reassuring that results from these different methods were consistent within sub-groups.

Limitations of our study included the cross-sectional design that precluded causal inference for factors associated with alcohol use, and the low response of women among casual workers. Responses to questions on reported alcohol use, type of alcohol consumed, amount and frequency of use and sexual behaviour may have been subject to social desirability bias. Drinking behaviour may have been underreported, but we can also not exclude that the amount of alcohol taken may have been exaggerated by some young people, e.g. out of a desire to impress interviewers. To minimise these types of bias, we ensured that interviewers and interviewees were of the same gender, that study staff were comparatively young themselves, and were trained to provide a friendly and conducive atmosphere during the interview. To minimize recall bias, we used the TLFB calendar and displays of standard alcoholic drinks.

We conclude that alcohol use is common among young people in northern Tanzania, that alcohol use is highest among college students and casual labourers, and that it is associated with a number of socio-demographic and structural factors some of which might be amenable to interventions. Apart from cultural influences such factors include alcohol use within the family, heavy exposure to alcohol advertisements and easy access to alcohol. Our study also supports observations from other studies that alcohol use may have adverse effects, including risky sexual behaviours. Alcohol use among young people is a significant public health problem in northern Tanzania and probably other parts of the country, particularly among college students and casual labourers. There is an urgent need for interventions to reduce hazardous alcohol use among young people. Such interventions should aim to address both individual factors for example, brief alcohol screening followed by motivational interviewing [39, 40] and structural level factors, and multi-sectoral responses may be needed involving the education and health sectors, but also tax regulation and changes in legislation.

## Supporting Information

S1 Fig This is the Recruitment flow chart among four groups of young people in northern Tanzania. (TIFF)

S1 File This is the Alcohol Epidemiology Questionnaire. (PDF)

S2 File This is the pictorial display of beers. (PDF)

S1 Table This is the Patterns of reported alcohol use using the Time Line Follow Back Calendar among young people who report alcohol use in the past 60 days in northern Tanzania. (PDF)

S2 Table This is the Description of factors for initiation and persistence of alcohol use among young people in northern Tanzania. (PDF)
