# Supplementary material for: The Epidemiology of Alcohol Use and Alcohol Use Disorders among Young People in Northern Tanzania
Source: PLoS One. 2015 Oct 7;10(10):e0140041. doi: 10.1371/journal.pone.0140041 (PMC4596556; doi:10.1371/journal.pone.0140041)
Supplement: S1 File — (PDF) [file pone.0140041.s002.pdf]

ALCOHOL EPIDEMIOLOGY QUESTIONNAIRE Study IDNO: [___|___|___|___|___]

SECTION 1: SOCIO-DEMOGRAPHIC INFORMATION

We begin this interview by asking you to provide basis information about yourself. This information is important and you are requested to provide as much details as possible.

QNo	Code	Questions and Filters	Coding Categories	Programming notes
101	Intdate	*Write date of interview*	|__|__| |__|__|__| |__|__|__|__| Day Month Year	
102a<br><br>102b	Location<br><br>Locationot	Location	Moshi Urban 1<br>Moshi Rural 2<br>Nyamagana 3<br>Sengerema 4<br>Other 5<br>(Specify)…………………	
103a<br><br>103b	Group<br><br>Groupother	Study group	Secondary school student 1<br>College student 2<br>university student 3<br>Employed in soft drink industry 4<br>Employed in a farm 5<br>Employed in a ginnery 6<br>Out of school-unstable employment 7<br>Other industry 8<br><br>(Specify)………………………………………….<br>.	
104	Sex	What is your gender?	Male 1<br>Female 2	
105	Dob	What is your date of birth?	|__|__| |__|__|__| |__|__|__|__| Day Month Year<br>***write 99 for day or 999 for month or 9999 for year if unknown***	
106	Age	How old are you?	|__|__| Write completed years	
107a<br><br>107b	Religion<br><br>Religionot	What is your religion?	Muslim 1<br>Roman Catholic 2<br>Africa Inland Church 3<br>Pentecostal 4<br>Lutheran 5<br>Other 6<br>(Specify)……………..………………..	

QNo	Code	Questions and Filters	Coding Categories	Programming notes
108a<br><br>108b	Tribe<br><br>Tribeot	What is your tribe?	Sukuma 1<br>Nyamwezi 2<br>Mkurya 3<br>Chagga 4<br>Pare 5<br>Haya 6<br>Other 7<br>(Specify)……………………………...	
109	Educat	What is the highest level of formal education you have completed?	Never went to school 1<br>Incomplete primary school 2<br>Complete primary school 3<br>Secondary (form one-four) 4<br>Secondary (form one-six) 5<br>University/ college 6	
110	Maristat	Currently, what is your marital status?	Single 1<br>Single (with boyfriend/girlfriend) 2<br>Cohabiting 3<br>Married 4<br>Separated/divorced 5<br>Widowed 6	
111	Income	On average, how much money do you have available each month through either your job or your family support?	TZS|__|__|__|__|__|__||__| ***(write 9999999 if don't know)***	
112a<br><br>112b	Live<br><br>liveother	Currently, where do you live?	I live at home with both parents 1<br>I live at home with one parent 2<br>I live at home with a relative/guardian 3<br>I live at home with my partner 4<br>I live at boarding school 5<br>I live in a hostel/rented room 6<br>I live in a rented apartment 7<br>Other 8<br>(Specify)……………………………...	
113	Pareducat	What is the highest level of education of the persons with whom you live?	Never went to school 1<br>Incomplete primary school 2<br>Complete primary school 3<br>Secondary (form one-four) 4<br>Secondary (form one-six) 5	

QNo	Code	Questions and Filters	Coding Categories	Programming notes
			University/ college 6<br>Unknown 7<br>Not applicable 8	

SECTION 2: ALCOHOL USE

In this section, we are going to ask you questions related to alcohol use. As you know there are many alcoholic drinks in the community and some people drink alcohol regularly. We would like to find out young people experiences with alcohol in this community. This information will remain confidential and will not be shared with other people. We are not here to judge you as a person but rather to gain understanding of a number of issues related to alcohol use among young people in this community. Remember we have not written your name on this form and this information will not be directly linked to you.

QNo	Code	Questions and Filters	Coding Categories	Skip instructions
201	alcoh1	In what year did you have your first alcoholic drink?	|__|__|__|__| Year<br>***write 9999 if unknown***<br>***write 0000 if never used alcohol***	If never used alcohol skip to Q206
202	alcoh2	How old were you when you had your first alcoholic drink?	|__|__| Years<br>***write 99 if unknown***	
203a<br><br>203b	Alcoh3<br><br>Alcoh3ot	At what occasion did you have your first alcoholic drink?	Holiday 1<br>Wedding ceremony 2<br>School party/graduation 3<br>At a part with friends 4<br>Other family cerebration 5<br>Other 6<br>(specify)………………………………..	
204a<br><br>204b	Alcoh4a<br>Alcoh4b<br>Alcoh4c<br>Alcoh4d<br>Alcoh4e<br>Alcoh4eot	What type of alcoholic drink did you drink?	No Yes<br>Bottled beer 1 2<br>Wine 1 2<br>Spirit/liquor 1 2<br>Local beer/spirit/liquor 1 2<br>Other 1 2<br>(Specify) …………………………	
205a<br><br>205b	Alcoh5a<br>Alcoh5b<br>Alcoh5c<br>Alcoh5d<br>Alcoh5e<br>Alcoh5eot	What were the reasons for drinking?<br>I wanted to try it<br>My friends convinced me<br>My parents/relatives advice me<br>My partner convinced me<br>Other<br>(specify)……………………………	No Yes<br>1 2<br>1 2<br>1 2<br>1 2<br>1 2	After this question skip to Q207

206a<br><br><br><br><br><br><br><br>206b	 Alcoh6a<br>Alcoh6b<br>Alcoh6c<br>Alcoh6d<br>Alcoh6e<br>Alcoh6f<br>Alcoh6fg<br>Alcoh6fot	What are the reasons for not starting/trying an alcoholic drink?<br>My parents are against alcohol<br>Relatives are against alcohol<br>My religion is against alcohol<br>My friends are against alcohol<br>I am afraid of alcohol side effects<br>Other<br>(Specify)………..…………………	No Yes<br>1 2<br>1 2<br>1 2<br>1 2<br>1 2<br>1 2<br>1 2	After this question skip to Q301
207	Alcoh7	In the past 12 months, have you ever drunk any alcohol?	No 1<br>Yes 2	If yes skip to Q209
208a<br><br><br><br><br><br><br><br><br><br><br><br>208b	 <br><br>Alcoh8a<br>Alcoh8b<br>Alcoh8c<br>Alcoh8d<br>Alcoh8e<br>Alcoh8f<br>Alcoh8g<br>Alcoh8h<br>Alcoh8i<br>Alcoh8ot	What are the reasons you didn't drink alcohol in the past 12 months?<br>My parents are against alcohol<br>Relatives are against alcohol<br>My religion is against alcohol<br>I have no money<br>I don't like alcohol<br>I'm home and not possible<br>I'm at college and not possible<br>I have health problems<br>Other<br>(Specify)…………..………………	No Yes<br>1 2<br>1 2<br>1 2<br>1 2<br>1 2<br>1 2<br>1 2<br>1 2<br>1 2	After this question skip to Q301
209a<br><br><br><br><br><br><br>209b	 <br>Alcoh9a<br>Alcoh9b<br>Alcoh9c<br>Alcoh9d<br>Alcoh9e<br><br>Alcoh9eot	What type of alcohol do you <u>usually drink</u>?<br>Bottled beer<br>Wine<br>Spirit/liquor<br>Local beer/spirit/liquor<br>Other<br>(Specify) ……………………	No Yes<br>1 2<br>1 2<br>1 2<br>1 2<br>1 2	
210	Alcoh10	<u>On average</u>, how many days in a week do you drink alcohol?	Number of days per week |___| ***(write 0 if none)(write 8 if do not drink on weekly basis)***<br>***(write 9 if unknown)***	

211	Alcoh11<br>Alcoh12	On average, how many drinks do you have on a typical day when you are drinking?	Indicate type of container used (e.g. beer bottles, glass of wine etc)<br>……………………………………………….<br>Amount of beverage |__|__|	
212	Audit1	How often do you have a drink containing alcohol?	Never 0<br>Monthly or less 1<br>2-4 times a month 2<br>2-3 times a week 3<br>4 or more times a week 4	
213	Audit2	How many standard drinks do you have on a typical day when drinking?	1 or 2 0<br>3 or 4 1<br>5 or 6 2<br>7 or 9 3<br>10 or more 4	
214	Audit3	How often do you have six or more drinks on one occasion?	Never 0<br>Less than monthly 1<br>Monthly 2<br>Weekly 3<br>Daily or almost daily 4	
215	Audit4	How often during the last year have you found that you were not able to stop drinking once you had started?	Never 0<br>Less than monthly 1<br>Monthly 2<br>Weekly 3<br>Daily or almost daily 4	
216	Audit5	How often during the last year have you failed to do what was normally expected of you because of drinking?	Never 0<br>Less than monthly 1<br>Monthly 2<br>Weekly 3<br>Daily or almost daily 4	
217	Audit6	How often during the last year have you needed a first drink in the morning to get yourself going after a heavy drinking session?	Never 0<br>Less than monthly 1<br>Monthly 2<br>Weekly 3<br>Daily or almost daily 4	
218	Audit7	How often during the last year have you had a feeling of guilt or remorse after drinking?	Never 0<br>Less than monthly 1<br>Monthly 2	

			Weekly 3<br>Daily or almost daily 4	
219	Audit8	How often during the last year have you been unable to remember what happened the night before because of your drinking?	Never 0<br>Less than monthly 1<br>Monthly 2<br>Weekly 3<br>Daily or almost daily 4	
220	Audit9	Have you or someone else been injured because of your drinking?	No 0<br>Yes, but not in the last year 2<br>Yes, during the last year 4	
221	Audit10	Has a relative, friend, doctor, or other health care worker been concerned about your drinking or suggested you cut down?	No 0<br>Yes, but not in the last year 2<br>Yes, during the last year 4	
222	dsm1	Have you ever failed to fulfill major role obligations at work, school, or home (e.g., repeated absences or poor work performance related to substance use; substance-related absences, suspensions or expulsions from school; or neglect of children or household)?	No 1<br>Yes, but not in the last year 2<br>Yes, during the last year 3	
223	dsm2	Do you drink when working, driving, or operating heavy machinery?	No 1<br>Yes, but not in the last year 2<br>Yes, during the last year 3	
224	dsm3	Have you ever gotten trouble with any authorities because you drank too much? (e.g. problems with school authorities, police etc)	No 1<br>Yes, but not in the last year 2<br>Yes, during the last year 3	
225	dsm4	Have you ever continued to drink alcohol despite persistent or recurrent social or interpersonal problems caused or exacerbated by the effects of the alcohol (e.g.,	No 1<br>Yes, but not in the last year 2<br>Yes, during the last year 3	

		arguments with spouse about consequences of intoxication or physical fights).		
226	dsm5	Have you ever felt a need to increase the amount of alcohol that you drink to achieve the desired effect which was initially attained by lesser amount of alcohol (few drinks)	No 1<br>Yes, but not in the last year 2<br>Yes, during the last year 3	
227	dsm6	Have you ever experienced a feeling of nervousness, feeling of shakiness, anxiety, irritability or easily excited, emotional volatility, rapid emotional changes and excessive sweating	No 1<br>Yes, but not in the last year 2<br>Yes, during the last year 3	
228	dsm7	Do you take alcohol (drug-valium) to get relief from the above experiences taking alcohol or valium?	No 1<br>Yes, but not in the last year 2<br>Yes, during the last year 3	
229	dsm8	Do you often consume alcohol in larger amounts over a longer period than was intended	No 1<br>Yes, but not in the last year 2<br>Yes, during the last year 3	
230	dsm9	Do you have a persistent desire or there are unsuccessful efforts to cut down or control alcohol use	No 1<br>Yes, but not in the last year 2<br>Yes, during the last year 3	
231	dsm10	Have ever felt that you spend a great deal of your time in activities necessary to obtain alcohol, use alcohol or recover from its effects	No 1<br>Yes, but not in the last year 2<br>Yes, during the last year 3	
232	dsm11	Have you reduced important social, occupational, or recreational activities are given up or reduced because of alcohol use	No 1<br>Yes, but not in the last year 2<br>Yes, during the last year 3	

SECTION 3: ALCOHOL ADDITIONAL INFORMATION

Thank you for providing responses to the alcohol questions so far this is highly appreciated. We have another set of questions to collect additional information related to alcohol. Please take a few minutes to complete this part. Your responses are very important and we hope to learn a lot from this information.

QNo	Code	Questions and Filters	Coding Categories	Programming notes
301	Advert1	During the past 30 days, how often did you see any alcohol advertisements?	Never 1<br>Rarely 2<br>Sometimes 3<br>Almost daily 4<br>Daily 5	
302	binge	During the past 30 days, how many times did you drink so much alcohol that you were really drunk?	0 times 1<br>1 or 2 times 2<br>3 to 9 times 3<br>10 or more times 4<br>Not applicable 5	
303	cost	During the past 30 days, how much of your money did you spend on alcohol drinks?	None 1<br>Less than Tshs 5000 2<br>Between Tshs 5,000-10000 3<br>More than Tshs 10,000 4<br>Not applicable 5	
304	costb	During the past 30 days, how much money was spent on alcohol, by others, offering you drinks?	None 1<br>Less than Tshs 5000 2<br>Between Tshs 5,000-10000 3<br>More than Tshs 10,000 4<br>Not applicable 5	
305	compl	During the past 30 days, how many times did you get into trouble with your family or friends, miss school, or get into fights as a result of drinking alcohol?	0 times 1<br>1 or 2 times 2<br>3 to 9 times 3<br>10 or more times 4<br>Not applicable 5	
306	whom	With whom do you usually drink alcohol?	With my friends 1<br>With my family 2<br>With persons I have just met 3<br>I usually drink alone 4<br>Not applicable 5	
307	relat	Do any of your brothers or sisters drink alcohol?	I do not have brothers/sisters 1<br>Yes 2<br>No 3	

			I do not know 4	
308	Influad1	When you watch television, videos, or movies, how often do you see actors drinking alcohol?	I do not watch TV, videos, or 1<br>movies 2<br>Never 3<br>Rarely 4<br>Sometimes 5<br>Most of the time 6<br>Always	
309	Influad2	When you go to sports events, fairs, concerts, community events, or social gatherings how often do you see advertisements for alcohol?	I do not go to these events 1<br>Never 2<br>Rarely 3<br>Sometimes 4<br>Most of the time 5<br>Always 6	
310	Influad8	If one of your best friends offered you a drink of alcohol, would you drink it?	Definitely not 1<br>Probably not 2<br>Probably yes 3<br>Definitely yes 4	
311	availab	How difficult do you think it would be for you to get alcohol, such as beer or wine? If you wanted to?	Impossible 1<br>Very difficult 2<br>Fairly difficult 3<br>Fairly easy 4<br>Very easy 5<br>I do not know 6	
312	Curric1	During the school years, were you taught in any of your classes about the problems associated with drinking alcohol?	No 1<br>Yes 2<br>I do not know 3	
313	Curric2	During the school years, were you taught in any of your classes about the effects of alcohol use on decision-making?	No 1<br>Yes 2<br>I do not know 3	
314	Curric3	During the school years, were you taught in any of your	No 1<br>Yes 2	

		classes about how to tell someone you did not want to drink alcohol?	I do not know 3	
315	Harma	Do you think that alcohol drinking may be harmful?	No 1<br>Yes 2<br>I do not know 3	*If the answer is No or I do not know skip to Q317*
316a<br><br>316b	Harmba<br>Harmbb<br>Harmbc<br>Harmbd<br>Harmbe<br>Harmbf<br>Harmbg<br>Harmbh<br>Harmbi<br>Harmbj<br>Harmbk<br>Harmbot	In general what are the possible problems with alcohol use?<br>Harmful to the liver<br>Harmful to the brain<br>Harmful to the stomach<br>May cause accidents/injuries<br>Makes people aggressive<br>Impair judgement<br>A person loose his/her job<br>Student may fail his/her studies<br>There are no problems<br>I don't know<br>Other<br>(specify)……………………………	No / Yes<br>1 2<br>1 2<br>1 2<br>1 2<br>1 2<br>1 2<br>1 2<br>1 2<br>1 2<br>1 2<br>1 2<br>1 2	
317	Atrisk	Do you think that you are at any risk because of your drinking habits?	No 1<br>Yes 2<br>I do not know 3<br>Not applicable 4	

SECTION 4: TOBACCO and other Drugs USE

I am now going to ask you about your experience in using tobacco and other drugs. These questions are important and we request you to respond as accurately as possible. Remember we are not here to judge you as a person. This information will remain confidential and will not be shared with other people.

QNo	Code	Questions and Filters	Coding Categories	Programming notes
401	tobdru1	Have you ever used any tobacco products in your life?	No 1<br>Yes 2	If No skip to Q405
402	tobdru2	Do you currently use any tobacco products?	No 1<br>Yes 2	If NO skip to Q405
403	tobdru3	Do you currently use tobacco products daily? (i.e., almost every day for at least one year)	No 1<br>Yes 2	
404	tobdru4	How old were you when you first started smoking daily? *(Probe age in years)*	|__|__|Years<br>***Write 99 if don't know***	
405	tobdru5	Have you ever smoked or used any drugs/substance in your life?	No 1<br>Ye 2<br>s	If No skip to Q501
406a		Which of these drugs/substances have you ever used?	No Yes	
	tobdru6a		Marijuana 1 2	
	tobdru6b		Heroin 1 2	
	tobdru6c		Cocaine 1 2	
	tobdru6d		Khat (mirungi) 1 2	
	tobdru6e		Other 1 2	
406b	tobdru6ot		(specify)………………………………	

SECTION 5: Sexual behavior

Thank you for responding to all these questions. This is the last section and I will ask information related to sexual behavior. I understand some of these questions may be sensitive. I request you to respond to as many questions as possible as this will be very helpful for this study.

QNo	Code	Questions and Filters	Coding Categories	Programming notes
501	sthiv1	How old were you when you first had sex?	|__|__|Years<br>***Write 99 if don't know***<br>***Write 88 for those who never had sex***	
502	Sthiv2	The last time you had sex, did you or your partner drink alcohol beforehand?	I have never had sex 1<br>Yes 2<br>No 3	
503	Sthiv3	The last time you had sex; did you or your partner use drugs beforehand?	I have never had sex 1<br>Yes 2<br>No 3	
504	Sthiv4	The last time you had sex, did you or your partner use a condom	I have never had sex 1<br>Yes 2<br>No 3	
505	Sthiv5	How often do you or your partner use a condom when you have sex?	I have never used condom 1<br>Rarely 2<br>Sometimes 3<br>Most of the time 4<br>Always 5<br>I have never had sex Never 6	
506	Sthiv6	How many different sex partners have you ever had?	I have never had sex 1<br>1 person 2<br>2 people 3<br>3 people 4<br>4 people 5<br>5 people 6<br>6 or more people 7<br>I do not remember 8	

507	Sthiv7	Have you ever been told by a doctor or nurse that you had a sexually transmitted infection, such as gonorrhoea, syphilis, HIV?	Yes 1<br>No 2<br>I do not know 3	
508	Sthiv8	Have you ever been tested for HIV infection or AIDS?	Yes 1<br>No 2	
509	Sthiv9	Have you ever experienced vaginal/penile discharge in the past 12 months	Yes 1<br>No 2<br>I do not know 3	
510	sthiv10	Have you ever had ulcers in your private parts (genitals) in the past 12 months	Yes 1<br>No 2<br>I do not know 3	
511	Sthiv11	In the past 12 months, how many different sex partners have you had?	|__|__|__| partners<br>***Write 999 if do not remember and 000 if did not sex in the past 12 months and 888 if never had sex***	
512	Sthiv12	In the last 30 days, how many casual sexual partners have had? (Causal partners are any sex partners who are not constant friend or partner; they may include people who you pay for having sex, or not)	None 1<br>One 2<br>More than one 3<br>I have never had sex 4	
513	sthiv13	Have you ever have sex under the influence of alcohol which you regretted the next day?	Yes 1<br>No 2<br>Don’t remember 3<br>I have never had sex 4<br>Not applicable 5	

Thank you for agreeing to participate in this study. I know this has taken a long time to finish. I know your participation have contributed to the success of this study. Let me know if you have any final questions or need any information related to the questions you were asked.

Data Reviewer	
Date	

	ENTRY 1	ENTRY 2
Initials		
Date		

ADDITIONAL INFORMATION DETERMINE TOTAL AMOUNT OF ALCOHOL INTAKE IN EACH DRINKING EVENT AS IDENTIFIED IN THE TFLB

Study ID: |___|___|___|___|___| **Event Number:|___|___|**

Thank you for agreeing to participate in our study and responding to earlier questions regarding alcohol use. I will now ask you some more question with the aim of describing further a particular drinking event.

**QNo.**	**Questions and Filters**	**Coding Categories**				**Codes & skip instructions**
101	Write interviewer's code or initials	INTERVIEWERCODE/ INITIALS [____|____]				staffenrq
102	Write date of interview	|__|__| |__|__|__| |__|__|__|__| DAY MONTH YEAR				dateenrol
103	Write date of the drinking event	|__|__| |__|__|__| |__|__|__|__| DAY MONTH YEAR				dateevent
	Drink name	Volume of the bottle/can	Number of bottles	Number of drinks	How many people shared the drink	
104a						drink1
104b						drink2
104c						drink 3
104d						drink 4
