# Supplementary material for: The Epidemiology of Alcohol Use and Alcohol Use Disorders among Young People in Northern Tanzania
Source: PLoS One. 2015 Oct 7;10(10):e0140041. doi: 10.1371/journal.pone.0140041 (PMC4596556; doi:10.1371/journal.pone.0140041)
Supplement: S2 File — (PDF) [file pone.0140041.s003.pdf]

Pictures of beers and standard drinks

LOCALLY AVAILABLE INDUSTRIAL MADE BEERS IN MWANZA, TANZANIA

Alcohol concentration expressed as Alcohol concentration by volume (ABV)

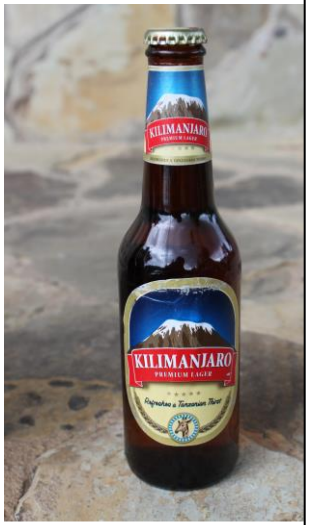

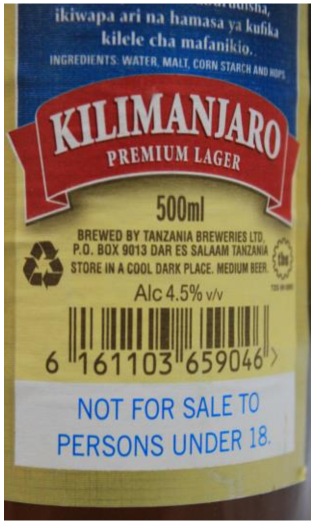

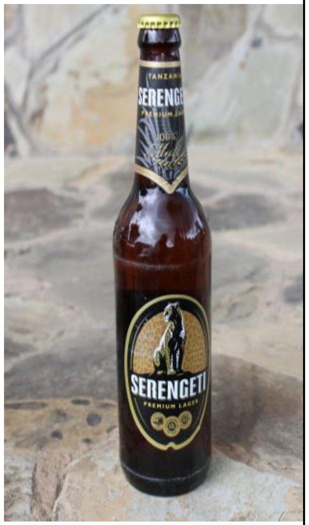

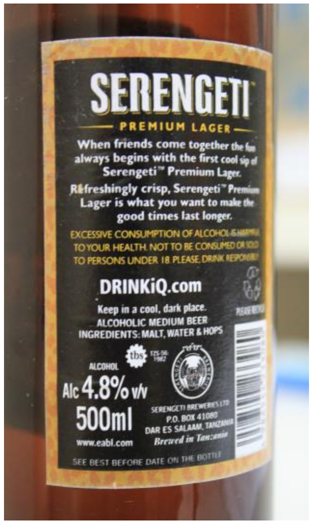

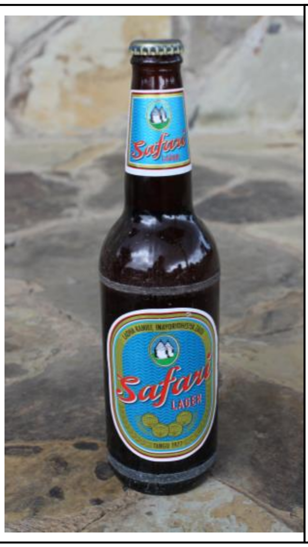

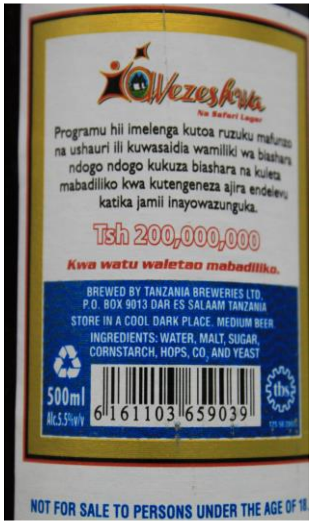

Kilimanjaro Premium Lager, 4.5% ABV, 500ml
(2 standard drinks)

Serengeti Premium Lager, 4.8% ABV, 500ml
(2 standard drinks)

Safari Lager, 5.5% ABV, 500ml
(3 standard drinks)

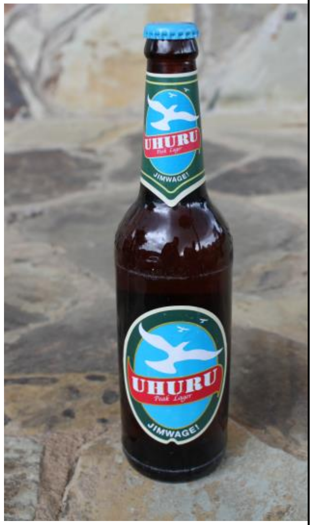

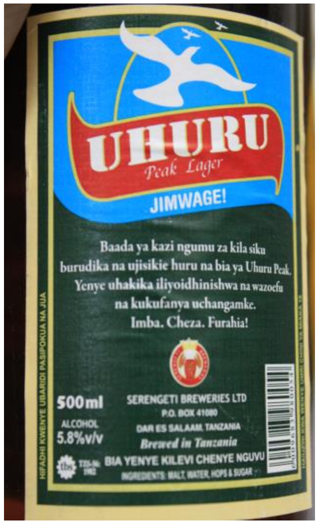

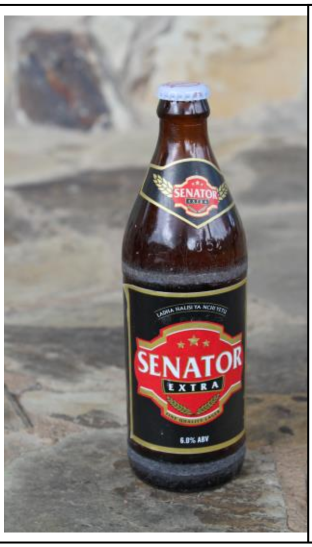

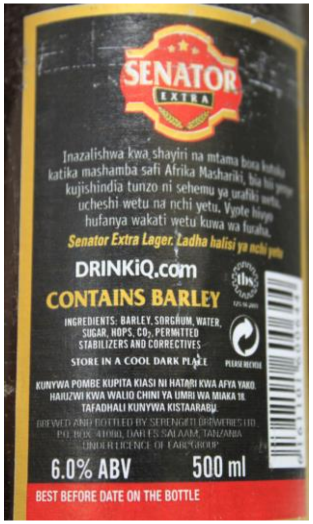

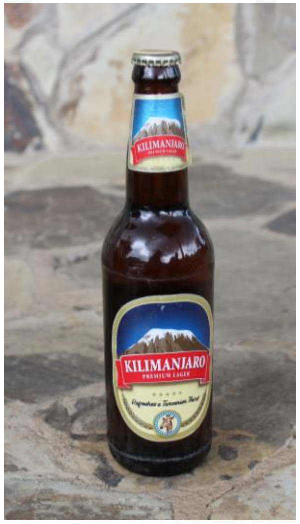

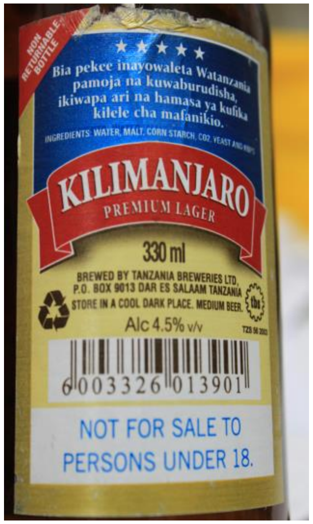

Uhuru Peak Lager, 5.5% ABV, 500ml
(3 standard drinks)

Senator Extra Lager, 6.0% ABV, 500ml
(3 standard drinks)

Kilimanjaro Premium Lager, 4.5% ABV, 330ml
(1 standard drink)

LOCALLY AVAILABLE INDUSTRIAL MADE BEERS IN MWANZA, TANZANIA

Alcohol concentration expressed as Alcohol concentration by volume (ABV)

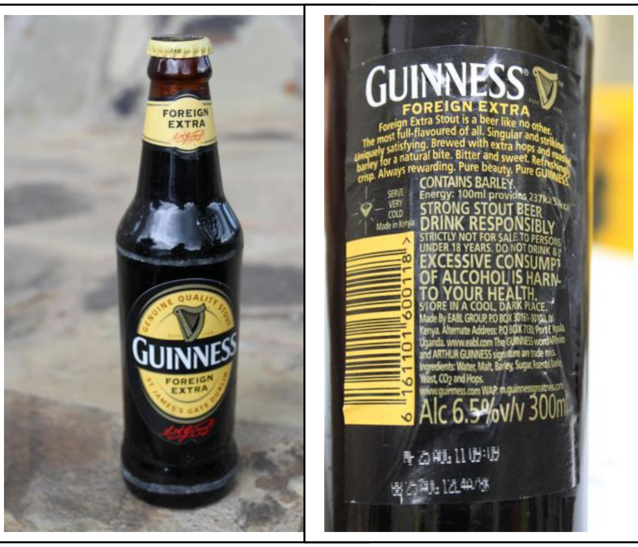

Guinness Foreign Extra, 6.5%ABV, 300ml
(2 standard drinks)

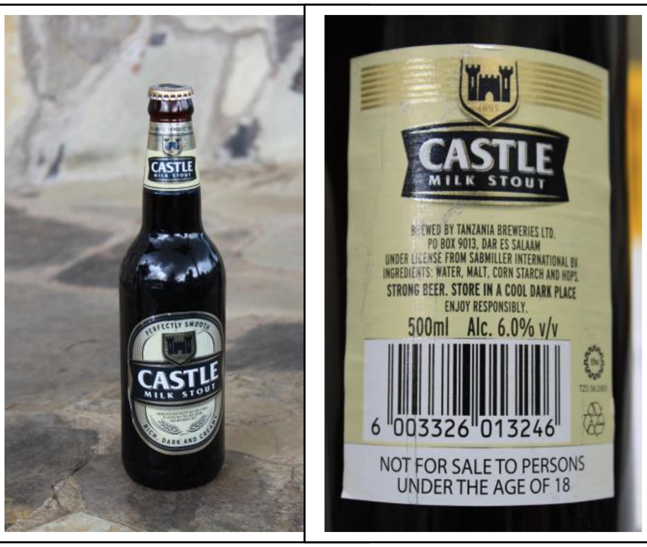

Castle Milk Stout, 6.0% ABV, 500ml
(3 standard drinks)

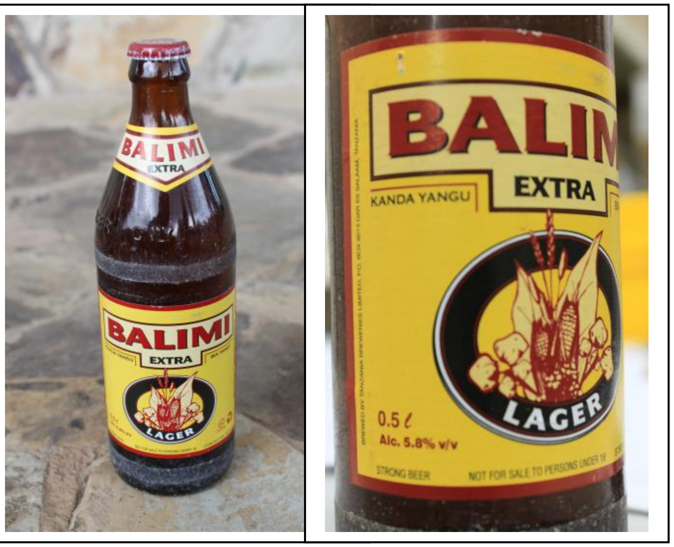

Balimi Extra, 5.8% ABV, 500ml
(3 standard drinks)

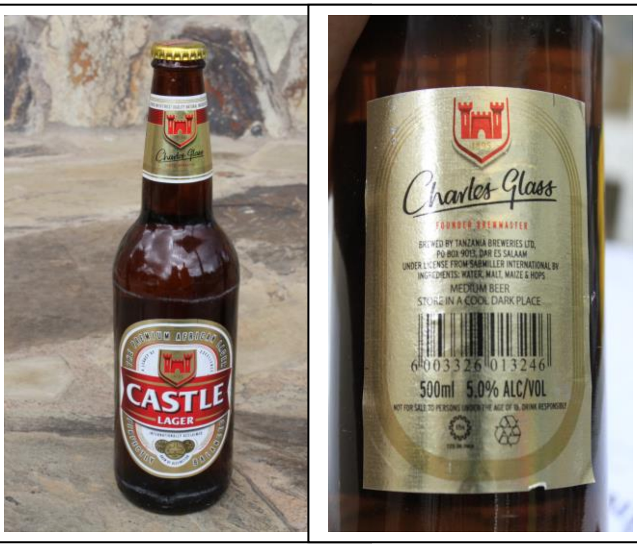

Castle Lager,5.0% ABV, 500ml
(3 standard drinks)

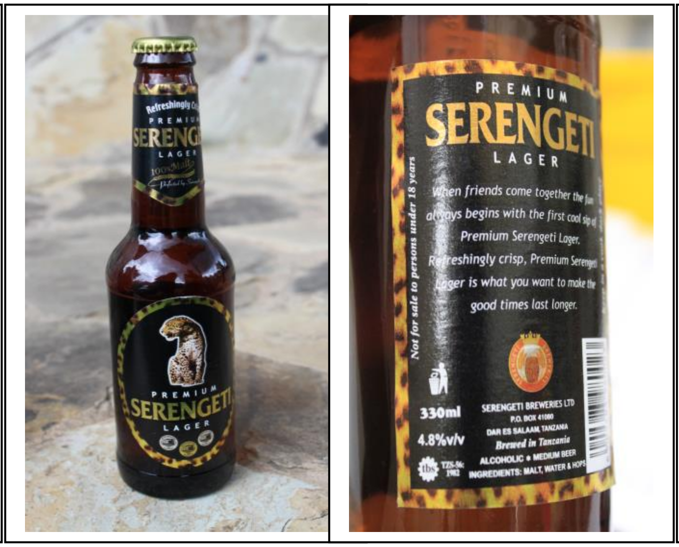

Serengeti Premium Lager, 4.8% ABV, 330ml
(2 standard drinks)

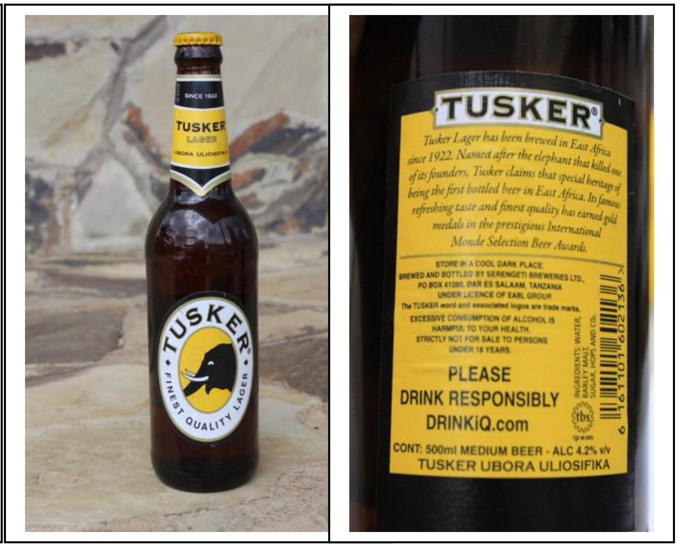

Tusker Lager, 4.2% ABV, 500ml
(2 standard drinks)

LOCALLY AVAILABLE INDUSTRIAL MADE BEERS IN MWANZA, TANZANIA

Alcohol concentration expressed as Alcohol concentration by volume (ABV)

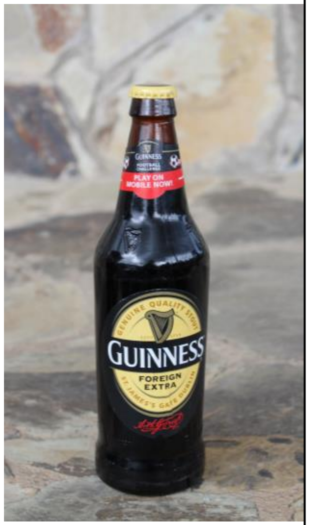

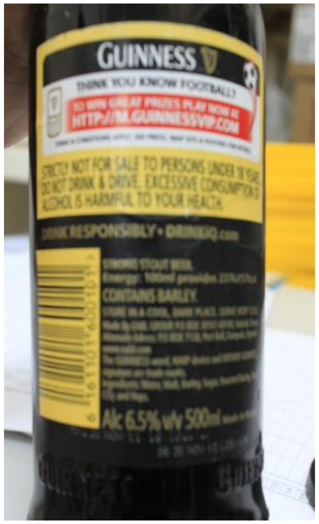

Guinness Foreign Extra, 6.5% ABV, 500ml
(3 standard drinks)

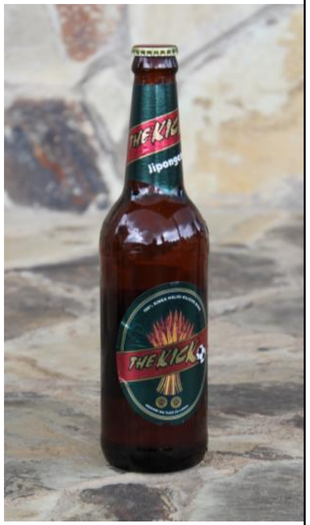

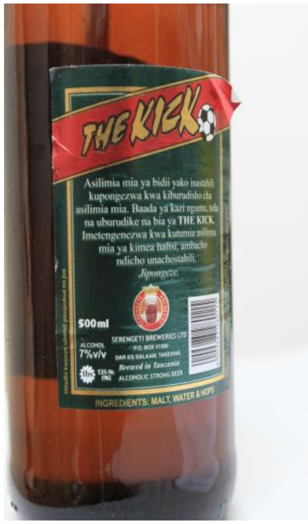

The Kick, 7.0% ABV, 500ml
(4 standard drinks)

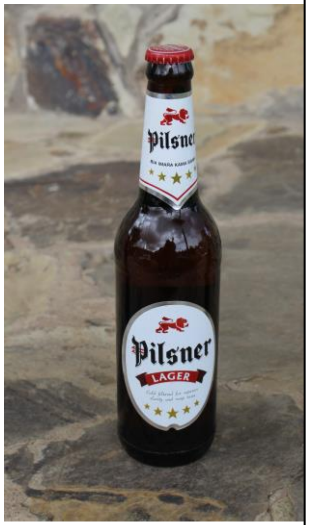

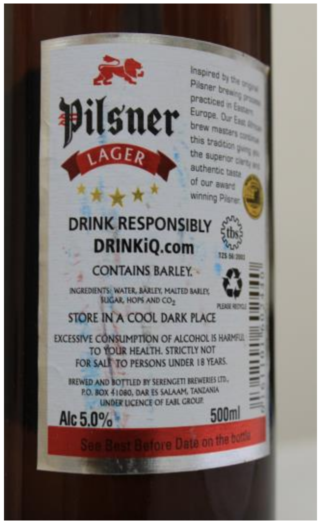

Pilsner Lager, 5.0% ABV, 500ml
(3 standard drinks)

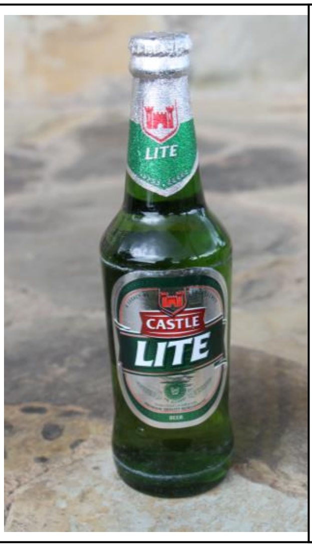

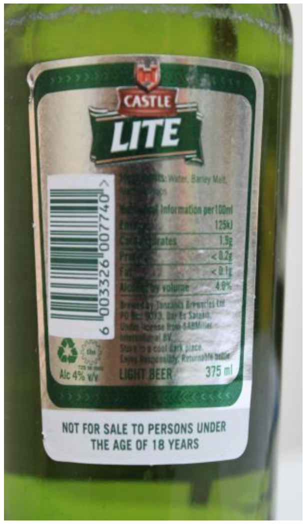

Castle Lite, 4.0% ABV, 375ml
(2 standard drinks)

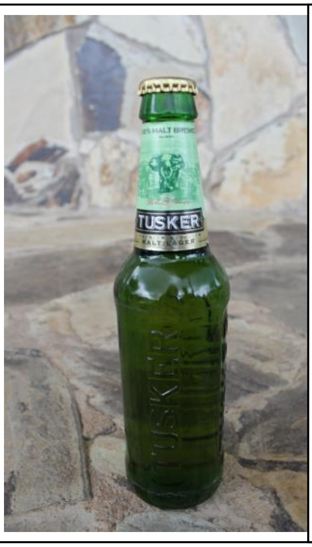

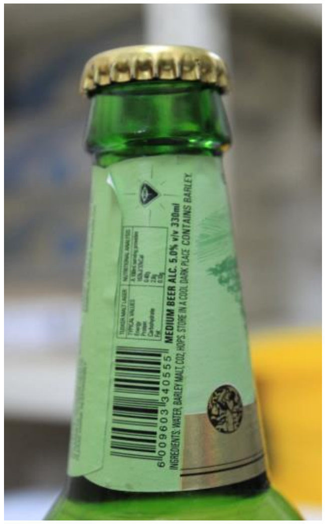

Tusker Malt Lager, 5.0% ABV, 330ml
(2 standard drinks)

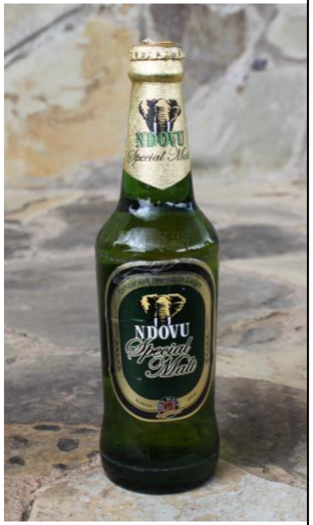

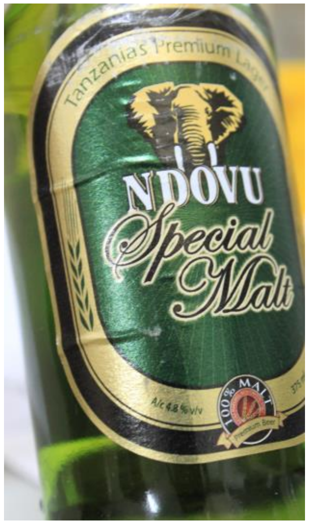

Ndovu Special Malt, 4.8% ABV, 375ml
(2 standard drinks)

LOCALLY AVAILABLE INDUSTRIAL MADE BEERS IN MWANZA, TANZANIA

Alcohol concentration expressed as Alcohol concentration by volume (ABV)

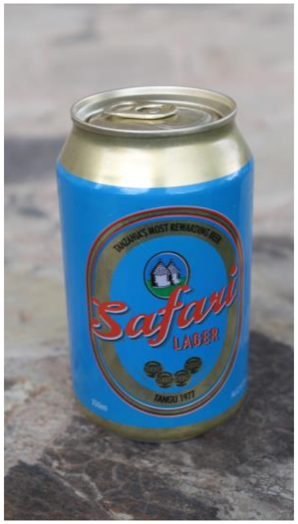

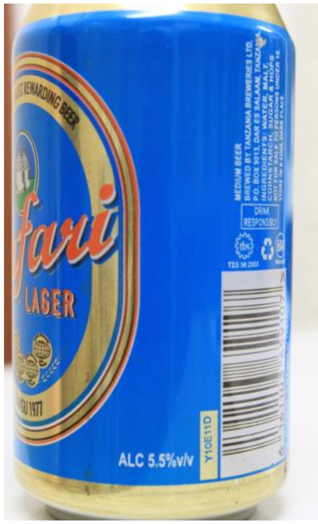

Safari Lager, 5.5% ABV, 330ml
(2 standard drinks)

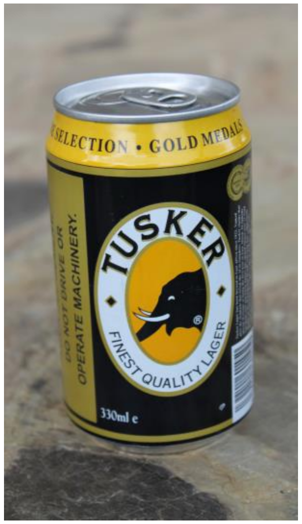

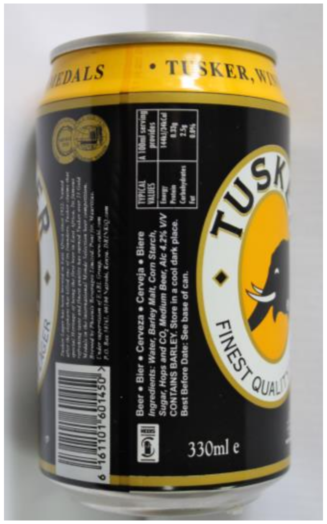

Tusker Lager, 4.2% ABV, 330ml
(1 standard drink)

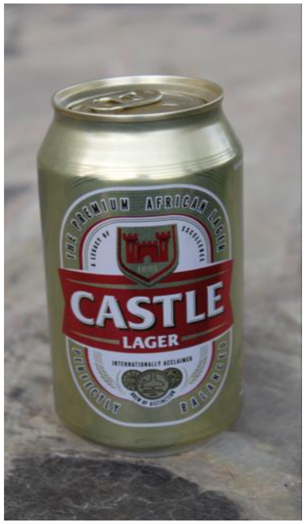

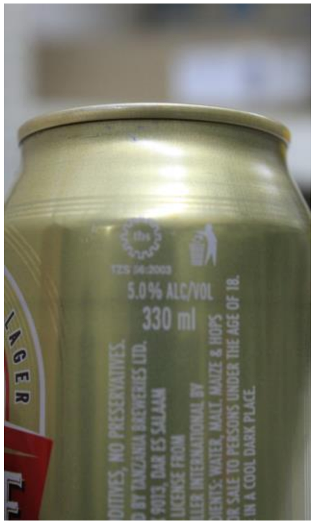

Castle Lager, 5.0% ABV, 330ml
(2 standard drinks)

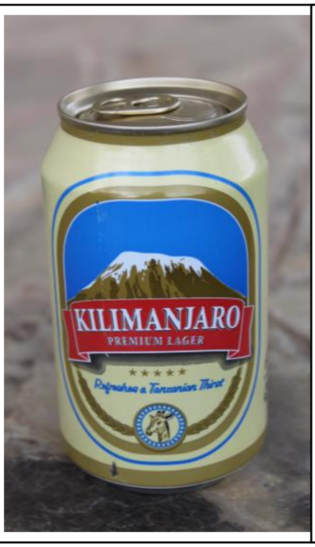

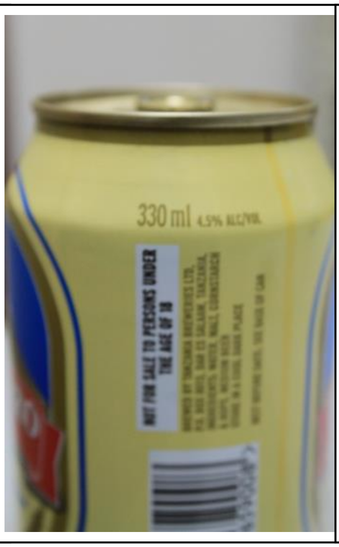

Kilimanjaro Premium Lager, 4.5% ABV, 330ml
(1 standard drink)

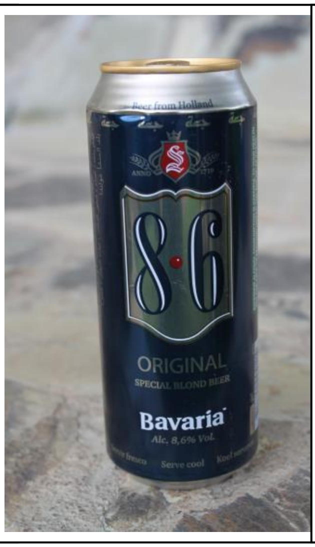

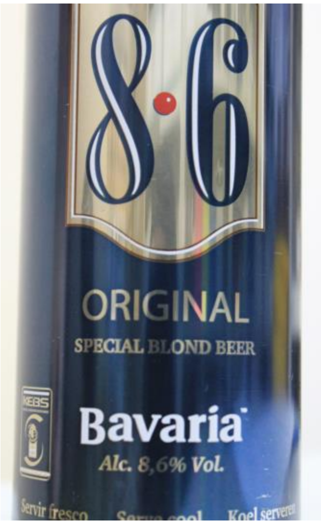

Bavaria Lager, 8.6% ABV, 500ml
(4 standard drinks)

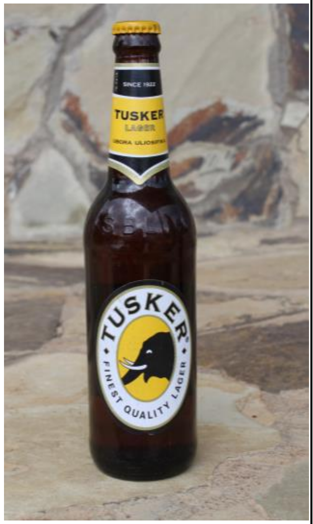

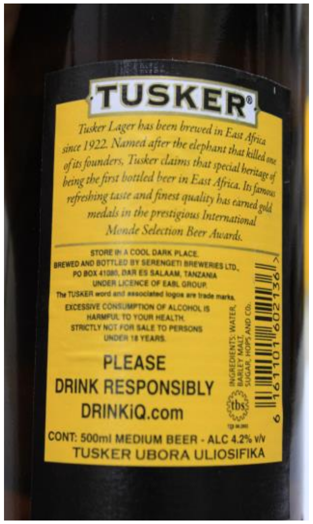

Tusker Lager,4,2% ABV, 500ml
(2 standard drinks)
