# Supplementary material for: The Epidemiology of Alcohol Use and Alcohol Use Disorders among Young People in Northern Tanzania
Source: PLoS One. 2015 Oct 7;10(10):e0140041. doi: 10.1371/journal.pone.0140041 (PMC4596556; doi:10.1371/journal.pone.0140041)
Supplement: S1 Table — (PDF) [file pone.0140041.s004.pdf]

**S1 Table: Patterns of reported alcohol use using the Time Line Follow Back Calendar among young people who report alcohol use in the past 60 days in northern Tanzania**

| | | Secondary school students[2] | | College and university students[2] | | Employed in local industries[3] | | Casual labourers[3] | |
|---|---|---|---|---|---|---|---|---|---|
| Variables | Responses | Female | Male | Female | Male | Female | Male | Female | Male |
| Sample | N[1] | 9 | 20 | 41 | 72 | 14 | 41 | 13 | 81 |
| **Time line follwoback calendar 60 days** | Events-median &IQR | 2[1-2] | 4[2-7] | 1[1-5] | 5[2-8] | 1[1-3] | 4[2-6] | 6[4-10] | 6[3-10] |
| | Drinks in 60 days-median &IQR | 2.5[1.3-9.0] | 20.0[3.5-49.0] | 6[2-36] | 39.8[12-62.6] | 0.8[0.3-12.0] | 21.0[6.0-44.0] | 18.0[8.0-30.0] | 40.8[12.5-73.6] |
| | Drinks in 30 days-median&IQR | 1.3[0.6-4.5] | 10.0[1.8-24.5] | 3[1-18] | 19.9[6-31.3] | 0.4[0.2-6.0] | 10.5[3.0-22.0] | 9.0[4.0-15.0] | 20.4[6.2-36.8] |
| | Drinks per event-median&IQR | 1.3[1.3-4.5] | 4.0[2.2-6.0] | 4.2[2.0-6.0] | 7.5[4.9-9.8] | 1.0[0.3-4.0] | 6.0[3.5-8.3] | 3.3[1.0-4.0] | 6.8[2.8-9.4] |
| | Days drinking in a month-median | 1[1-1] | 2[1-4] | 1[1-3] | 2[1-4] | 1[1-2] | 2[1-3] | 3[2-5] | 3[2-5] |
| | Heavy episodic drinking[4] (%, 95%CI) | 7.5[0-25.4] | 26.7[0.0-33.0] | 26.3[14.9-55.0] | 71.2[51.0-91.1] | 7.1[0.0-25.0] | 51.2[36.7-66.7] | 7.7[0.0-8.3] | 56.8[47.8-71.2] |

. [1]Actual number of respondents without sampling weights applied. [2]Median are weighted estimates. [3]Median without sampling weights applied. [4]Heavy episodic drinking is defined as average of 6 or more standard drinks in a drinking occasion
