# Supplementary material for: The Epidemiology of Alcohol Use and Alcohol Use Disorders among Young People in Northern Tanzania
Source: PLoS One. 2015 Oct 7;10(10):e0140041. doi: 10.1371/journal.pone.0140041 (PMC4596556; doi:10.1371/journal.pone.0140041)
Supplement: S2 Table — (PDF) [file pone.0140041.s005.pdf]

S 2 Table: Description of factors for initiation and persistence of alcohol use among young people in northern Tanzania

		Secondary school students[4]		College and university students[4]		Employed in local industries[5]		Casual labourers [5]	
Variables	Responses	Female	Male	Female	Male	Female	Male	Female	Male
Sample	N[1]	63	101	111	169	58	152	23	272
**First drinking occasion among ever users**	Holiday(festivity/recreation)	23.6[18.4-28.6]	20.1[16.6-25.8]	22.4[17.1-28.3]	21.9[17.6-24.2]	22.4[13.7-37.9]	30.9[26.2-36.0]	26.1[0.0-30.8]	37.5[33.3-41.2]
	Wedding	3.8[0.0-7.8]	13.4[4.0-29.2]	15.7[7.3-18.5]	9.2[0.8-10.9]	8.6[2.8-16.0]	7.2[2.8-12.5]	17.4[0.0-50.0]	8.1[5.0-10.7]
	School party	6.9[0.0-11.8]	1.2[0.0-3.7]	5.2[4.5-7.5]	19.1[8.2-25.2]	6.9[0.0-11.2]	2.6[0.6-5.1]	4.3[0.0-5.1]	2.9[0.8-5.1]
	Party with friends	7.6[1.7-13.8]	3.0[0.0-7.9]	7.7[6.0-10.3]	6.5[0.4-7.6]	6.9[0.0-18.6]	4.6[1.3-7.9]	8.7[0.0-100]	4.0[1.5-6.6]
	Other family celebration	17.3[7.3-32.7]	33.6[31.0-36.8]	13.5[10.3-24.6]	11.5[9.8-11.8]	20.7[11.0-37.0]	16.4[11.0-22.2]	17.4[0.0-20.5]	16.2[10.7-19.9]
	Not a special occasion	25.9[16.7-34.8]	23.4[3.6-35.4]	20.7[18.5-31.4]	25.7[16.0-43.4]	22.4[8.7-28.4]	32.9[26.0-39.0]	21.7[0.0-25.6]	23.2[18.9-28.8]
	Other	7.2[2.4-12.0]	3.1[1.0-7.6]	5.4[2.7-7.2]	2.2[0.7-3.0]	3.4[0.0-11.1]	4.6[1.0-8.0]	4.3[0.0-100.0]	4.8[2.3-8.8]
**Drink at first occasion among ever users**	Bottled beer	30.9[12.1-43.6]	40.5[20.5-48.5]	59.7[56.6-69.9]	66.0[52.0-68.8]	51.7[39.0-69.0]	55.3[48.0-63.2]	34.8[21.1-100.0]	47.8[38.8-60.9]
	Wine	4.9[0.0-12.5]	1.0[0.5-1.8]	19.9[7.5-24.2]	7.5[0.9-9.8]	6.9[0.0-16.7]	1.3[0.0-3.0]	0.0	0.0
	Spirit/liqour	5.6[0.5-10.0]	13.0[7.1-15.7]	1.9[0.0-10.8]	7.4[4.7-12.3]	0.0	11.8[7.3-17.0]	0.0	10.7[7.7-15.6]
	Local beer/spirit/liquor	44.5[30.5-66.4]	31.8[17.8-67.2]	13.8[6.1-16.2]	17.7[16.6-23.8]	36.2[20.0-48.1]	27.6[18.1-37.4]	39.1[0.0-46.2]	31.6[18.2-39.4]
	Other	1.6[0.0-4.2]	0.0	0.0	0.0	0.0	0.0	0.0	0.0
	More than one type	12.6[10.4-14.9]	13.7[2.8-20.4]	4.6[1.0-16.2]	1.5[0.1-9.1]	5.2[0.0-10.3]	3.9[0.0-7.7]	26.1[0.0-30.8]	9.9[6.3-12.2]
**Reason for drinking at first occasion among ever users**	Wanted to try	51.8[44.7-58.0]	43.9[41.6-48.6]	33.0[27.1-49.6]	48.8[36.4-53.8]	44.8[31.5-61.5]	36.2[29.1-44.5]	60.9[0.0-100.0]	35.3[31.5-39.6]
	Convinced by a friend	0.4[0.2-0.7]	18.9[15.4-22.4]	2.5[0.0-9.7]	16.3[1.4-24.6]	0.0	17.1[10.0-24.2]	0.0	12.9[8.8-19.8]
	Advice from parents/relatives	4.1[0.0-8.5]	8.1[4.2-11.0]	14.2[1.3-16.5]	5.0[3.2-9.5]	3.4[0.0-8.2]	7.2[1.8-11.3]	0.0	3.3[1.0-6.3]

	Convinced by a partner					0.0	0.7[0.0-1.9]	0.0	0.4[0.0-0.6]
	other	0.1[0.0-0.4]	0	1.1[0.2-6.7]	2.3[0.7-8.9]	1.7[0.0-5.1]	1.3[0.0-3.8]	0.0	1.5[0.0-3.9]
	more than one reason	43.6[33.5-54.1]	29.1[25.0-33.6]	49.2[42.9-50.4]	27.6[17.0-48.9]	50.0[35.8-64.1]	37.5[23.9-48.7]	39.1[0.0-100.0]	46.7[36.1-53.1]
	N[2]	198	155	152	93	68	148	20	174
**Reasons for not drinking among those never used alcohol**	Parents	91.4[87.4-93.9]	91.0[87.0-95.8]	73.3[67.3-76.5]	85.7[77.4-89.8]	76.9[65.7-88.1]	65.0[58.6-73.2]	44.4[25.0-100.0]	74.7[68.5-80.5]
	Relatives	74.9[69.7-78.5]	51.5[41.6-67.4]	40.5[36.4-48.1]	45.9[43.2-52.4]	52.3[35.9-67.3]	51.4[42.5-60.2]	11.1[0.0-100.0]	60.0[53.2-67.2]
	Religion	59.6[54.0-66.0]	69.5[60.3-77.7]	67.1[60.4-70.3]	92.8[78.0-98.7]	60.0[49.3-79.8]	62.9[52.0-70.5]	33.3[0.0-100.0]	68.2[64.1-72.8]
	Friends	60.1[55.9-63.1]	39.9[32.6-49.6]	31.6[28.5-38.0]	38.3[26.7-43.1]	43.1[26.3-56.0]	37.9[26.7-46.0]	11.1[0.0-100.0]	33.5[26.0-42.3]
	Afraid of side effects	91.6[88.3-94.3]	93.1[90.9-95.4]	92.7[90.8-96.8]	97.6[91.6-100.0]	95.4[91.8-100.0]	92.1[84.5-97.2]	88.9[81.8-100.0]	91.2[87.1-95.2]
	N[3]	30	53	47	69	28	91	3	142
**Reasons for not drinking in the past 12 months among ever users**	Parents	60.2[38.9-81.4]	78.6[71.6-85.4]	63.3[47.2-66.8]	45.9[41.5-63.9]	60.7[25.8-72.4]	50.5[37.0-61.3]	66.7[0.0-100.0]	66.2[59.6-73.0]
	Relatives	51.5[38.0-61.7]	45.9[35.0-65.9]	17.3[15.1-24.0]	25.6[19.1-34.8]	28.6[0.0-37.1]	26.4[16.7-34.3]	33.3[0.0-100.0]	45.1[37.5-53.5]
	Religion	34.5[1.8-58.7]	62.9[57.0-73.1]	53.9[45.9-63.9]	44.8[28.7-76.1]	42.9[23.1-66.1]	57.0[46.2-66.0]	66.7[0.0-100.0]	51.4[40.3-57.6]
	I have no money	30.6[13.9-47.9]	4.7[2.0-7.9]	2.2[0.0-9.4]	5.5[0.0-8.4]	0.0	4.0[0.0-7.9]	0.0	5.6[2.9-8.7]
	Dislike alcohol	67.8[55.7-80.9]	85.4[81.5-91.8]	85.0[64.6-91.3]	99.2[96.0-100.0]	85.7[71.4-100.0]	87.9[79.8-93.8]	66.7[0.0-100.0]	87.3[78.8-94.0]
	Being at home	30.7[16.8-43.0]	22.2[7.8-41.2]	5.1[0.0-21.2]	2.0[0.0-9.9]	17.9[0.0-33.3]	6.6[1.6-10.2]	0.0	5.6[0.5-8.1]
	Being at college	17.9[12.3-25.3]	23.2[18.2-35.1]	1.6[0.0-9.3]	7.3[1.9-17.6]	0.0	0.0	0.0	0.0
	I have health problems	7.0[0.0-16.8]	9.5[6.0-17.6]	6.0[0.0-8.0]	4.8[0.0-14.0]	7.1[0.0-16.0]	6.6[3.2-10.8]	0.0	10.6[6.6-14.0]
	I am afraid of side effect	12.0[0.0-28.0]	23.0[15.0-33.9]	6.8[2.3-27.4]	35.1[14.3-46.3]	10.7[0.0-33.3]	29.7[23.5-37.6]	33.3[0.0-100.0]	23.9[19.0-34.2]
	Other	13.5[4.0-20.8]	9.3[0.0-22.5]	12.1[7.8-13.5]	6.1[0.7-17.3]	21.4[10.1-60.8]	24.2[12.9-34.4]	33.3[0.0-100.0	19.0[12.8-23.8]

[1] Actual number of respondents ever used alcohol without sampling weights applied. [2]Actualnumber of respondents never used alcohol without sampling weights applied. [3] Actual number of respondents drunk alcohol in the last year among ever users without sampling weights applied. [4]Proportions are weighted estimates. [5]Proportions without sampling weights applied.
